# State-dependent facial pulsation asymmetry and phase asynchrony measured by imaging photoplethysmography and their coupling with contingent negative variation in migraine

**DOI:** 10.3389/fneur.2026.1818649

**Published:** 2026-06-04

**Authors:** Huanyu Li, Qinghua He, Qingru Chang, Yongxiang Zhang, Qiuxia Deng, Zhiyuan Sun, Yunbo Fu, Fei Yin, Yudan Lv

**Affiliations:** 1 Department of Neurology and Neuroscience Center, The First Hospital of Jilin University, Changchun, China; 2 State Key Laboratory of CNS/ATM and MIIT Key Laboratory of Complex-field Intelligent Sensing, Beijing Institute of Technology, Beijing, China; 3 Guangdong Province Key Laboratory of Intelligent Detection in Complex Environment of Aerospace, Land and Sea, Beijing Institute of Technology, Zhuhai, China; 4 Department of Neurology, Beijing Tiantan Hospital, Headache Center, Capital Medical University, Beijing, China; 5 Changchun Institute of Optics, Fine Mechanics and Physics, Chinese Academy of Sciences, Changchun, Jilin, China; 6 Department of Neurology, The Second Hospital of Jilin University, Changchun, China

**Keywords:** central–peripheral coupling, contingent negative variation, facial microcirculation, imaging photoplethysmography, migraine

## Abstract

**Objective:**

To characterize imaging photoplethysmography (iPPG)-derived facial hemodynamic alterations across migraine states and examine their association with contingent negative variation (CNV), an electroencephalography-derived marker of anticipatory cortical activity.

**Methods:**

We enrolled 78 patients with migraine, including 60 assessed during the interictal phase (IP) and 18 assessed during the migraine attack phase (MAP), together with 72 healthy controls (HC). Short facial videos were analyzed across three facial angiosomes: the lateral forehead, mid-forehead, and cheek. Two peripheral pulsation metrics were derived: bilateral pulsation amplitude asymmetry (BPA) and bilateral pulsation phase difference (BPP). CNV recordings obtained during an S1–S2 paradigm were used to quantify initial CNV (iCNV), overall CNV (oCNV), and terminal CNV (tCNV) amplitudes and areas. Group effects and CNV–iPPG associations were evaluated using linear mixed-effects models adjusted for age and sex. Sensitivity analyses evaluated potential confounding by mood symptoms, headache impact, acute medication use, and unequal IP–MAP sample sizes. Exploratory receiver operating characteristic analyses were used to assess within-cohort separability before and after adjustment for age and sex.

**Results:**

BPA and BPP were higher in patients with migraine than in HC across facial angiosomes. CNV metrics showed state-dependent differences: IP participants had higher iCNV, oCNV, and tCNV amplitudes and areas than both HC and MAP participants, whereas MAP participants were generally comparable to HC. CNV amplitudes were positively associated with BPA and BPP, with angiosome-dependent slopes and stronger group differences in iCNV-related models. Exploratory analyses showed high within-cohort separability for mid-forehead iPPG metrics in distinguishing migraine from HC and for central CNV metrics in distinguishing MAP from IP.

**Conclusion:**

Migraine is associated with increased facial pulsation asymmetry and phase asynchrony, state-dependent CNV alterations, and angiosome- and state-dependent central–peripheral coupling. These candidate markers require validation in larger, longitudinal, and external cohorts.

## 1 Introduction

Migraine is a common primary headache disorder characterized by recurrent attacks of moderate to severe pulsating pain (1–3). It is often accompanied by nausea, photophobia, and phonophobia, and is typically aggravated by routine physical activity (4). Migraine can substantially impair social functioning and quality of life and imposes a sustained healthcare and socioeconomic burden. Currently, clinical diagnosis and disease monitoring rely largely on subjective information, including medical history, questionnaires, and headache diaries. These approaches have limited ability to continuously capture prodromal and ictal phases and their transient physiological fluctuations in real-world settings (5, 6). In recent years, objective neurophysiological and neuroimaging modalities, such as electroencephalography (EEG), somatosensory evoked potentials (SEPs), and functional magnetic resonance imaging (fMRI), have provided important insights into altered cortical excitability regulation and dysfunctional brain networks in migraine (7–9). They have also been explored for diagnostic support, state stratification, and the assessment of treatment response and prognosis. However, their use is constrained by equipment requirements, cost, and operational complexity, which limits feasibility for continuous monitoring and broad implementation in daily practice. Therefore, developing objective, reproducible, accessible, and scalable migraine-related markers is essential for subtype assessment and dynamic disease management (10–12).

Accumulating evidence suggests that migraine cannot be explained by a purely “vascular” or “neural” mechanism. Instead, it involves multi-level processes including activation of the trigeminovascular system, neurogenic inflammation, and vascular responses mediated by several vasoactive transmitters (13–15). Within this framework, calcitonin gene-related peptide (CGRP) is a key vasoactive neuropeptide. It modulates vascular tone and can influence local perfusion and hemodynamic responses, and is considered a central component of migraine pathophysiology (16, 17). Accordingly, perfusion and pulsation features of superficial facial vessels may provide a non-invasive and repeatable peripheral window in migraine by capturing inter-side amplitude asymmetry and phase asynchrony derived from pulsation amplitude and phase across facial angiosomes, thereby characterizing state-dependent differences in neurovascular regulation (18, 19).

Recent advances in optical imaging and signal processing have enabled non-invasive and potentially continuous assessment of blood perfusion and vascular pulsation features (20–22). However, most existing work has focused on estimating vital signs such as heart rate, respiratory rate, and blood pressure (23–25). Fewer studies have systematically characterized migraine-related spatial heterogeneity in hemodynamic responses, for example differences in inter-side pulsation amplitude asymmetry and phase asynchrony within distinct vascular territories. Therefore, we implemented a territory-based model across facial angiosomes and quantified vascular pulsation features within each territory. This approach aims to more sensitively capture state-dependent, spatially heterogeneous hemodynamic alterations in migraine and to improve clinical interpretability.

Imaging photoplethysmography (iPPG) is a non-invasive optical technique that has received increasing attention in recent years (26, 27). It uses a camera to acquire continuous skin images and converts pixel-level intensity changes into time-series signals. Cardiac-cycle driven fluctuations in subcutaneous blood volume induce subtle variations in reflected light intensity. After signal processing, pulsatile components can be extracted to obtain pulse-wave information that is comparable to conventional contact-based photoplethysmography (PPG). Compared with contact PPG measured at a single site, iPPG can capture pulsatile signals over a wide field of view and across multiple facial angiosomes. This enables spatially resolved assessment of local hemodynamic responses and is well suited to investigating potential regional differences in migraine. Although iPPG is sensitive to illumination conditions and facial motion, methodological progress in motion suppression, illumination correction, and territory-based modeling has improved signal reproducibility and physiological interpretability (28–30). These advances support scalable measurements in real-world settings.

Contingent negative variation (CNV) is a slow negative cortical potential that develops during the interval between a warning cue and an imperative stimulus and is generally considered to reflect anticipatory attention, expectancy, and motor preparation (31–33). In migraine, CNV has long been regarded as one of the most extensively studied electrophysiological markers, because it provides a non-invasive window into higher-order information processing and cortical preactivation (33, 34). Prior studies have reported several CNV abnormalities in migraine, including larger negative interictal amplitudes and altered habituation patterns, which have been interpreted as reflecting abnormal cortical information processing and excitability regulation between attacks (35–37). Importantly, CNV abnormalities may vary across the migraine cycle. Several studies have reported that the larger negative amplitudes and reduced habituation observed interictally are attenuated during the attack or in the postattack period, in some cases approaching healthy-control levels (36, 38). However, recent reviews have also highlighted substantial heterogeneity across CNV studies in migraine, indicating that observed findings may vary according to paradigm design, component definition, habituation measures, and patient characteristics (37, 39, 40). Against this background, it remains unclear whether central preparatory activity indexed by CNV is systematically coupled with peripheral vascular pulsation features in migraine, or whether such coupling is reorganized across clinical states.

To address this gap, we used iPPG to extract pulsation features from distinct facial angiosomes in healthy controls and individuals with migraine. Specifically, we quantified bilateral absolute amplitude difference and bilateral absolute phase difference to capture inter-side amplitude asymmetry and phase asynchrony within each angiosome. Key parameters of CNV were quantified concurrently in the same session. We aimed to achieve three objectives. First, we characterized superficial facial hemodynamic features and cortical slow potential features across migraine clinical states, including the interictal and ictal phases. Second, within a linear mixed-effects modeling framework that accounts for repeated measurements across facial angiosomes, we tested the coupling pattern between CNV and peripheral pulsation features and evaluated its state dependence. This analysis was designed to provide quantitative evidence consistent with altered coordination between central preparatory activity and peripheral vascular pulsation in migraine. Third, we conducted exploratory receiver operating characteristic analyses to illustrate within-cohort separability for migraine versus healthy controls and for the ictal versus interictal phase, with the goal of informing future model development for state monitoring and stratification. Overall, this study proposes an interpretable and scalable framework that jointly profiles peripheral facial hemodynamics and central electrophysiology, thereby expanding objective evidence for neurovascular dysregulation in migraine.

## 2 Methods

### 2.1 Participants

We enrolled 78 patients with migraine who attended the outpatient neurology clinic at the First Hospital of Jilin University between March and September 2025. Of these, 60 were assessed during the interictal phase (IP) and 18 during the migraine attack phase (MAP). During the same period, 72 healthy controls (HC) were recruited from the hospital health examination center and community volunteers. HC had no history of migraine, no chronic pain, and no known neurological or psychiatric disorders. They were not taking medications that could materially affect central nervous system activity or vascular tone and autonomic responses, including sedative hypnotics, antiepileptic drugs, antipsychotics, antidepressants, and beta-blockers. Demographic characteristics are summarized in Table 1. The study protocol was approved by the Ethics Committee of the First Hospital of Jilin University, and all participants provided written informed consent.

**Table 1:** Demographic characteristics and migraine-related clinical features across study groups.

Variable	HC (*n* = 72)	IP (*n* = 60)	MAP (*n* = 18)	Statistic	*p* value
Age, years	44.00 ± 10.42	44.30 ± 10.44	41.72 ± 9.57	F = 0.45	0.641
BMI, kg/m^2^	23.21 ± 2.32	23.53 ± 2.35	23.41 ± 2.49	*F* = 0.31	0.735
Female sex, *n* (%)	46(63.9)	39(65.0)	12(66.7)	χ^2^ = 0.05	0.974
Education level, *n* (%)				χ^2^ = 0.84	0.991
Primary school	11(15.3)	9(15.0)	2(11.1)		
Middle school	29(40.3)	25(41.7)	7(38.9)		
High school	14(19.4)	12(20.0)	3(16.7)		
College or above	18(25.0)	14(23.3)	6(33.3)		
Migraine-related clinical features (patients only)					
Time since diagnosis, months, *n* (%)	—			χ^2^ = 5.65	0.062
<6	—	22(36.7)	11(61.1)		
6–11	—	19(31.7)	6(33.3)		
≥12	—	19(31.7)	1(5.6)		
Monthly headache days (MHD), days	—	10.00(6.00, 14.00)	10.00(6.00, 14.25)	Z = −0.14	0.896
Pain intensity (VAS)	—	7.00(6.00, 7.00)	7.00(6.00, 7.00)	Z = 0.22	0.823
HIT-6 total score	—	52.00(49.00, 55.00)	55.00(53.00, 59.25)	Z = −2.67	0.007
PSQI total score	—	7.00(6.00, 8.00)	7.00(6.00, 9.00)	Z = −0.46	0.643
HAMA total score	—	8.00(7.00, 8.75)	8.00(7.75, 10.50)	Z = −2.10	0.031
HAMD total score	—	4.00(3.00, 6.00)	7.00(6.00, 8.00)	Z = −3.75	<0.001

Data are presented as mean ± SD for approximately normally distributed continuous variables, median (IQR) for non-normally distributed continuous variables, and n (%) for categorical variables. For three-group comparisons (HC, IP, and MAP), one-way ANOVA was used for normally distributed continuous variables, the Kruskal–Wallis test was used for non-normally distributed continuous variables, and Pearson’s chi-square test was used for categorical variables. When expected cell counts were insufficient, Fisher’s exact test or a Monte Carlo exact approach was applied as appropriate. For patient-only comparisons (IP vs MAP), an independent-samples *t*-test was used for normally distributed variables (t statistic reported), and the Mann–Whitney U test was used for non-normally distributed variables (Z statistic reported). “Time since diagnosis” was analyzed as a categorical variable using Pearson’s chi-square test, with p values estimated by a fixed-margin Monte Carlo exact method (20,000 simulations). All tests were two-sided, and *p* < 0.05 was considered statistically significant. BMI, body mass index; HAMA, Hamilton Anxiety Rating Scale; HAMD, Hamilton Depression Rating Scale; HC, healthy controls; HIT-6, 6-item Headache Impact Test; IP, interictal phase; IQR, interquartile range; MAP, migraine attack phase; MHD, monthly headache days; PSQI, Pittsburgh Sleep Quality Index; VAS, visual analog scale.

Patients with migraine met the following inclusion criteria: (1) diagnosis of migraine without aura according to the International Classification of Headache Disorders, 3rd edition (ICHD-3), no other primary headache disorders such as tension-type headache or cluster headache; (2) age 18 to 65 years, ability to understand and complete the study procedures; and (3) right-handedness. Exclusion criteria were (1) secondary headache or an identifiable structural cause; (2) a history of or current major neurological disease such as intracerebral hemorrhage, traumatic brain injury, or brain tumor; (3) significant hearing impairment that could affect task compliance; (4) use of centrally acting medications that could substantially affect EEG performance or task execution prior to acquisition, including sedative hypnotics, antipsychotics, and benzodiazepines; (5) use of preventive migraine medications within the previous 3 months or initiation, discontinuation, or dose adjustment of such medications before acquisition, including beta-blockers, calcium-channel blockers, antiepileptic drugs, antidepressants, and CGRP-pathway preventives; (6) substance or alcohol misuse; and (7) inability to complete the study protocol.

To minimize acute autonomic and hemodynamic perturbations, participants were instructed to refrain from caffeine and nicotine intake prior to acquisition. Headache phase was determined based on symptoms at the time of acquisition and verified against headache diaries. The MAP was defined as the presence of an ongoing headache attack at acquisition, with pain intensity recorded along with the time since attack onset. The IP was defined as no headache at acquisition, more than 24 h since the end of the previous attack, and more than 24 h before the onset of the subsequent attack, as verified retrospectively using subsequent diary entries to reduce potential prodromal effects. All participants documented headache status at acquisition, which was cross-checked bidirectionally with diary records. Because acute symptomatic treatment is common in outpatient migraine populations, including nonsteroidal anti-inflammatory drugs (NSAIDs), occasional or non-regular NSAID use was not an exclusion criterion to reduce selection bias. Acute medication use before acquisition was recorded, including medication class and, when available, the timing of last dose and dose amount.

We collected demographic information from all participants, including age, sex, body mass index, and educational attainment. Patients with migraine completed structured questionnaires and provided headache diaries documenting headache characteristics, disease history, and medication use. In patients, headache burden was assessed using monthly headache days (MHD). Pain intensity was rated using a visual analog scale (VAS). Headache-related disability was assessed using the 6-item Headache Impact Test (HIT-6). Sleep quality was evaluated using the Pittsburgh Sleep Quality Index (PSQI). Anxiety and depressive symptoms were assessed using the Hamilton Anxiety Rating Scale (HAMA) and the Hamilton Depression Rating Scale (HAMD), respectively. Because affective symptom scores and headache-related impact may differ between the ictal and interictal phases, we prespecified sensitivity analyses within the patient subgroup. Specifically, HAMA and HAMD were included as covariates to evaluate potential confounding by mood symptoms, whereas HIT-6 was included to evaluate whether headache-related impact materially influenced the IP–MAP comparisons. Details are provided in the statistical analysis section.

### 2.2 Facial angiosome framework

Our territory-based analysis of migraine-related facial vascular pulsation was grounded in the angiosome concept (41, 42). The angiosome model proposes that skin and soft tissue can be partitioned into relatively distinct anatomical perfusion territories defined by their source arteries. Although adjacent angiosomes are interconnected through anastomoses and perfusion can be redistributed via collateral pathways such as choke vessels, each territory is predominantly supplied by its corresponding source artery under physiological conditions (43). This provides a rationale for expecting partially distinct hemodynamic and pulsation characteristics across territories. In the face, soft tissue perfusion is mainly supplied by the external carotid artery system and its branches, including the superficial temporal artery and the facial artery. However, specific regions may receive contributions from branches of the internal carotid artery system via the ophthalmic artery. For example, the central forehead and periorbital regions can be supplied by the supraorbital and supratrochlear arteries (44–46). From a pathophysiological perspective, activation of the trigeminovascular system is closely linked to migraine pain and accompanying autonomic vascular responses. This activation may induce dynamic changes in vascular tone and perfusion in both intracranial and extracranial vessels (13, 15, 47). Given the anatomical heterogeneity in arterial supply, vascular responses related to migraine may also vary across facial angiosomes (48–50). Therefore, aggregating the whole-face signal into a single summary measure may obscure territory-specific effects and reduce physiological interpretability.

Accordingly, we adopted an anatomically guided territory-based strategy to extract facial hemodynamic signals. The face was first partitioned into standardized subregions and then regrouped according to source-artery territories to derive anatomically interpretable, territory-specific pulsation metrics. Based on extracranial vascular territories relevant to migraine, we prespecified three paired facial angiosomes for analysis. The lateral forehead primarily reflects the superficial temporal artery territory. The mid-forehead reflects forehead regions that may receive contributions from the ophthalmic artery via the supraorbital and supratrochlear arteries. The cheek primarily reflects the facial artery territory. These territories represent distinct patterns of arterial supply and provide an anatomical framework for comparing state-dependent peripheral pulsation features in migraine and for interpreting their cross-system association with central preparatory electrophysiological measures.

### 2.3 Acquisition and processing of vascular pulsation signals

**Figure 1:**
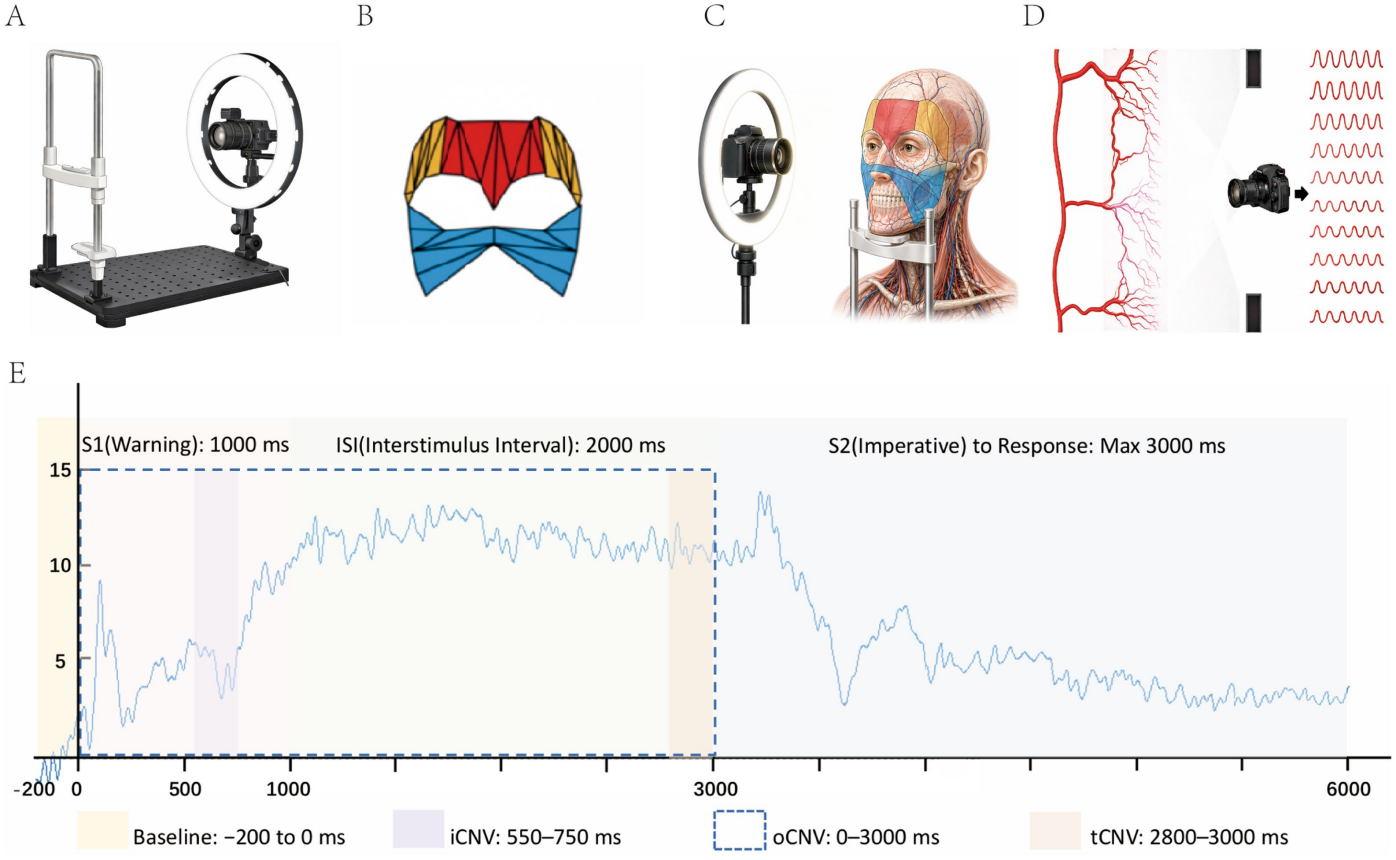
Schematic overview of the trans-angiosome imaging photoplethysmography (TaiPPG) framework and CNV assessment. **(A)** Schematic of the trans-angiosome imaging photoplethysmography (TaiPPG) acquisition setup. A 16 bit high-dynamic-range camera and a ring-shaped LED light source were used with orthogonal polarization to reduce specular reflection. A chin rest stabilized head position to minimize motion artifacts during non-contact facial video acquisition. **(B)** Enlarged angiosome-based facial mask set used for territory-specific segmentation and analysis. Three paired facial angiosomes were defined, including the lateral forehead (yellow), mid-forehead (red), and cheek (blue). **(C)** Schematic illustration of facial video acquisition with the angiosome-based mask mapped onto the face after facial alignment. This panel shows how the predefined facial angiosomes were used for territory-specific iPPG segmentation. **(D)** Overview of iPPG signal processing and hemodynamic feature extraction. Cardiac-related components were extracted from the facial videos and summarized within each facial angiosome to derive territory-specific pulsation waveforms, from which bilateral pulsation amplitude asymmetry (BPA) and bilateral pulsation phase difference (BPP) were computed. **(E)** CNV task paradigm and representative waveform. A 1,000 ms auditory warning cue (S1) was presented at 0 ms. The interval from S1 offset to S2 onset (interstimulus interval; ISI) was 2,000 ms; therefore, the S1-onset-to-S2-onset stimulus onset asynchrony was 3,000 ms. The imperative stimulus (S2) was presented at 3,000 ms and remained on until response, with a maximum duration of 3,000 ms. Electroencephalography (EEG) signals were baseline-corrected using the −200 to 0 ms window preceding S1 onset. CNV metrics were quantified as initial contingent negative variation (iCNV, 550–750 ms), overall contingent negative variation (oCNV, 0–3,000 ms), and terminal contingent negative variation (tCNV, 2,800–3,000 ms). For visualization and statistical consistency, the representative CNV waveform is shown after sign inversion, such that larger positive values indicate greater negative CNV magnitude.

Facial vascular pulsation videos were acquired using a trans-angiosome imaging photoplethysmography (TaiPPG) system (Figures 1A–D). In this study, TaiPPG refers to a territory-based facial imaging photoplethysmography framework that combines non-contact facial video acquisition with predefined facial angiosome masks to extract angiosome-resolved pulsation amplitude and phase features. The system consisted of a 16-bit high-dynamic-range camera (Chronos 1.4) and a ring-shaped white LED light source. A zoom lens (Computar 12.5–75 mm, f/1.2) was used to image facial reflectance onto the camera sensor. To reduce specular reflections from the skin surface, linear polarizers were placed in front of the lens and the light source with orthogonal orientations. Illumination uniformity was quantified using U1 and U2 metrics (U1 = 0.99, U2 = 0.95), indicating relatively uniform lighting across the acquisition field. To minimize large motion artifacts, a chin rest was used to stabilize head position. Before formal acquisition, participants sat quietly for 30 s to stabilize heart rate, and room temperature and humidity were kept as constant as feasible. Each acquisition consisted of a 5 s facial video recorded at 100 fps, with a spatial resolution of 640 × 512 pixels for the R, G, and B channels. Subsequent analyses used the green channel because it provides a higher signal-to-noise ratio for pulsatile signals among the three channels (51).

Videos were processed offline to recover cardiac-related pulsation patterns. We applied a lock-in amplification approach to extract heart-rate–related components from the video sequence. Specifically, within each predefined time window corresponding to the 5 s recording, the dominant heart-rate frequency was estimated using a fast Fourier transform of a whole-face perfusion signal derived from spatial averaging. This frequency was then used to construct a reference function. Pixel-wise perfusion signals were demodulated using lock-in detection to selectively preserve and enhance components at the reference frequency while suppressing other frequency components. This procedure improves the signal-to-noise ratio of pulsation waveforms obtained from short recordings (52). Under the assumption that cardiac-phase dynamics remain approximately stationary within a short time window, the lock-in output provides pixel-level cardiac-related components that serve as the basis for subsequent territory-based spatial integration and time–frequency feature extraction. The lock-in pulsation amplitude reflects periodic changes in pixel reflectance intensity. Because absolute calibration was not performed, amplitude was reported in arbitrary units (a.u.). To improve comparability across participants, amplitude was intensity-normalized during feature construction, as described in the subsequent section on feature definition and normalization.

To enable territory-specific analysis, we constructed a facial angiosome mask set based on the angiosome framework. As illustrated in Figure 1B, the three paired facial angiosomes were operationalized as an angiosome-based mask set for subsequent territory-specific iPPG segmentation, and Figure 1C illustrates how this mask was mapped onto the face after facial landmark alignment. An 81-point facial landmark detection algorithm was used to align individual faces, after which regions prone to micro-movement artifacts, including the periorbital and perioral areas, were excluded from subsequent analysis. The face was then partitioned into 36 elementary triangular units, which were regrouped according to extracranial arterial supply territories to form six side-specific angiosome masks. These were further organized into three paired facial angiosomes consistent with the present study design, namely the lateral forehead, mid-forehead, and cheek. For signal extraction, the predefined angiosome mask was applied after facial landmark alignment, and cardiac-related pixel-level signals within each facial angiosome were spatially integrated to derive a representative pulsation waveform for each angiosome (53). This anatomically guided mask set enabled consistent and repeatable territory-based signal extraction across participants and recording conditions. By relying on predefined anatomical territories rather than identifiable individual facial texture features, this approach enhanced analytical robustness while preserving participant privacy.

To characterize the dynamic pulsation patterns within facial angiosomes in the time–frequency domain, each representative pulsation waveform derived from lock-in reconstruction was subjected to continuous wavelet transform (CWT), yielding a complex-valued time–frequency spectrum $W_{s,k}(f,t)$ . Here, s∈{L,R} denotes the left and right sides, and k represents the three angiosome groups: lateral forehead, mid-forehead, and cheek.

The complex spectrum was decomposed into amplitude and phase components:


$$A_{s,k}(f,t)=|W_{s,k}(f,t)|,\phi_{s,k}(f,t)=∠W_{s,k}(f,t)$$

where amplitude reflects pulsation intensity and phase reflects relative timing.

Within the heart rate–related frequency band F=[0.8,1.8] Hz, which covers the typical resting heart rate range and encompasses the dominant frequency estimated during lock-in processing, amplitude values were averaged across frequencies to obtain a time-resolved amplitude series:


$${\overset{ˉ}{A}}_{s,k}(t)=\mathrm{mea}n_{f\in F}\;A_{s,k}(f,t)$$

Because lock-in and CWT-derived amplitudes originate from periodic variations in pixel reflectance intensity and were not absolutely calibrated, values were expressed in arbitrary units. To improve comparability across participants and reduce scaling effects related to skin reflectance, camera gain, and illumination variability, amplitude time series were intensity-normalized:


$${\tilde{A}}_{s,k}(t)=\frac{{\overset{ˉ}{A}}_{s,k}(t)}{\mathrm{mea}n_{t}({\overset{ˉ}{A}}_{s,k}(t))}$$

This normalization preserves within-participant inter-side relative structure while removing global intensity scaling differences.

Bilateral pulsation amplitude asymmetry (BPA) was defined for each angiosome as the time-averaged absolute difference between normalized left and right amplitudes:


$$\mathrm{BP}A_{k}=\mathrm{mea}n_{t}(|{\tilde{A}}_{L,k}(t)-{\tilde{A}}_{R,k}(t)|)$$

For phase analysis, inter-side phase difference was first calculated:


$$\Delta \phi_{k}(f,t)=\phi_{L,k}(f,t)-\phi_{R,k}(f,t)$$

To enhance phase stability and avoid bias introduced by circular wrap-around, complex averaging across the frequency band was performed:


$${\overset{ˉ}{\Delta \phi }}_{k}(t)=∠(\mathrm{mea}n_{f\in F}e^{\mathrm{j\Delta}\phi_{k}(f,t)})$$

Bilateral pulsation phase difference (BPP) was then defined as the time-averaged absolute circular phase difference:


$$\mathrm{BP}P_{k}=\mathrm{mea}n_{t}(|\text{wrapToPi}({\overset{ˉ}{\Delta \phi }}_{k}(t))|)$$

where wrapToPi confines phase values to the interval [−π,π] . After normalization, BPA is dimensionless, whereas BPP is expressed in radians.

All recordings were obtained using a fixed 5-s acquisition window. BPA and BPP were computed as time averages rather than cycle-integrated metrics to reduce direct scaling effects from inter-individual heart rate variability. To ensure signal reliability, each video segment underwent quality inspection. Recordings were excluded if facial landmark registration failed, motion artifacts were visually apparent, or angiosome-level pulsation signals could not be reliably reconstructed due to insufficient signal-to-noise ratio. BPA and BPP were calculated only for recordings that passed quality control. Because the present study focuses on metric construction and cross-system association analyses, detailed proportions of specific exclusion categories are not reported here but will be incorporated in future pre-registered validation studies.

### 2.4 CNV acquisition and processing

CNV recordings were obtained in a quiet, sound-attenuated room at a comfortable ambient temperature. Participants were seated comfortably, instructed to remain relaxed, and asked to minimize head and facial movements. The warning cue (S1) consisted of an auditory stimulus presented at approximately 1,000 Hz and 75 dB SPL, with a duration of approximately 1,000 ms. The imperative stimulus (S2) was an auditory stimulus of the same nominal type and intensity and remained present until the participant responded by pressing a button, with a maximum duration of 3,000 ms. Participants were instructed to maintain attention after S1 onset and to respond as quickly as possible following S2 onset by pressing a button. The interstimulus interval (ISI) between S1 offset and S2 onset was fixed at 2000 ms; therefore, S2 onset occurred 3,000 ms after S1 onset. A maximum response window of 3,000 ms was allowed after S2 onset. Electroencephalography (EEG) data were segmented time-locked to S1 onset (0 ms), covering the 200 ms pre-S1 baseline, the S1–S2 interval, and the post-S2 response window from −200 to 6,000 ms relative to S1 onset (Figure 1E).

EEG was recorded using Ag/AgCl electrodes placed according to the international 10–20 system. The left and right mastoids were used as reference, and data were sampled at 500 Hz. Electrode impedances were kept below 5 kΩ. CNV preprocessing was performed in MATLAB using EEGLAB (v2022.1). Continuous EEG was band-pass filtered at 0.1–30 Hz. Segments with gross artifacts, such as prominent movement-related contamination, were removed by visual inspection. Independent component analysis (ICA) was then applied to identify and remove artifact-related components. Epochs were time-locked to S1 onset and baseline-corrected using the mean voltage from −200 to 0 ms. Artifact-free trials were averaged to obtain subject-level CNV waveforms. Based on the temporal evolution of CNV, we derived three CNV metrics: initial contingent negative variation (iCNV), overall contingent negative variation (oCNV), and terminal contingent negative variation (tCNV), computed within predefined scalp regions (frontal, fronto-central, and central electrode groups). iCNV was defined as the early CNV component within a fixed 200 ms window from 550 to 750 ms after S1 onset. The mean amplitude and area under the curve (AUC) within this window were calculated as the iCNV amplitude and area, respectively. oCNV was defined as the overall preparatory negative shift from S1 onset to immediately before S2 onset; therefore, the mean amplitude and AUC from 0 to 3,000 ms after S1 onset were calculated as the oCNV amplitude and area, respectively. tCNV was defined as the terminal preparatory activity immediately before S2 onset; the mean amplitude and AUC within the 200 ms window preceding S2 onset, namely 2,800–3,000 ms after S1 onset, were calculated as the tCNV amplitude and area. For ease of interpretation and between-group comparison, CNV is a negative-going component. We therefore reported CNV measures as absolute values after sign inversion, such that larger values indicate a larger negative CNV amplitude or area.

### 2.5 Central–peripheral association analysis

To examine cross-system associations between peripheral hemodynamic features and central preparatory slow potentials across migraine states, we organized the data in a central–peripheral coupling framework. Peripheral measures were derived from three paired facial angiosomes, including the lateral forehead, mid-forehead, and cheek. For each angiosome, we quantified bilateral pulsation amplitude asymmetry (BPA) and bilateral pulsation phase difference (BPP). Thus, each participant contributed repeated peripheral measurements across the three angiosome groups. This repeated-measures structure motivated the use of a linear mixed-effects modeling framework, which is well suited for data in which multiple observations are nested within the same participant and within-participant correlation is modeled through participant-specific random effects (54–56). Central measures were CNV amplitudes computed from prespecified scalp electrode groups in the CNV paradigm. Because the central scalp region represents the canonical topographic maximum of CNV and typically provides higher signal-to-noise ratio and better cross-participant stability, central-region CNV amplitudes were used as the primary central predictors. This strategy also reduced the number of comparisons and mitigated the risk of chance findings due to multiple testing. Because CNV exhibits a stage-dependent temporal evolution, we *a priori* selected iCNV and oCNV amplitudes as representative phase metrics to improve physiological interpretability while limiting model multiplicity. The iCNV indexed early preparation following S1, whereas oCNV indexed sustained preparatory activity across the full S1-to-S2 interval. Within the prespecified analysis plan, the BPA–iCNV association was designated as the primary analysis. The BPA–oCNV association and all BPP-based models were designated as prespecified secondary analyses. These secondary analyses were used to evaluate phase dependence of the association pattern (iCNV versus oCNV) and feature-dimension dependence (BPA versus BPP). As a targeted sensitivity analysis for the central–peripheral coupling models, HAMA and HAMD were additionally included as covariates within the patient subgroup (IP and MAP), because affective symptoms may plausibly influence both central preparatory activity and peripheral hemodynamic responses. We compared the key CNV interaction terms and stratified association slopes before and after this adjustment to determine whether the coupling findings were materially altered. Detailed sensitivity analyses are described in the Statistical Analysis section.

### 2.6 Receiver operating characteristic (ROC) analysis

To assess the ability of peripheral pulsation features and central CNV metrics to discriminate between groups, we performed exploratory receiver operating characteristic (ROC) analyses for two binary classification tasks. ROC curves and the AUC were used as standard measures of within-cohort separability and were interpreted according to established diagnostic test evaluation frameworks (57–59). The first task compared migraine (IP and MAP combined) with HC. The second task compared MAP with IP within the patient cohort. Candidate single predictors included BPA and BPP derived from the three paired facial angiosomes (lateral forehead, mid-forehead, and cheek), as well as central-region iCNV and oCNV amplitudes. ROC analyses were conducted at the subject level. Each participant contributed one BPA and one BPP value for each angiosome and one value each for iCNV and oCNV. No frame-level analyses and no repeated-measures modeling were used for ROC estimation. Given the limited MAP sample size and the number of candidate predictors examined, these ROC analyses were prespecified as exploratory. They were intended to quantify within-cohort discriminative effect sizes and associated uncertainty, rather than to establish definitive diagnostic thresholds for clinical use. No internal resampling-based validation or external validation was performed, and the reported AUCs may therefore be optimistic estimates of out-of-sample performance.

### 2.7 Statistical analysis

Continuous variables are summarized as mean ± standard deviation or median (interquartile range), depending on distribution. Categorical variables are reported as n (%). The primary analyses included three groups (HC, IP, and MAP). Patient-only comparisons and all sensitivity analyses were restricted to the patient subgroup (IP and MAP). Group differences in peripheral pulsation metrics were evaluated using linear mixed-effects models (LMMs) according to established random-effects modeling frameworks for repeated-measures data. BPA and BPP were analyzed separately as dependent variables. Group (HC, IP, and MAP) was specified as a between-subject fixed effect, and facial angiosome (lateral forehead, mid-forehead, cheek) was specified as a within-subject fixed effect. The group-by-angiosome interaction was tested. Participant-specific random intercepts were included to account for within-subject correlation across angiosomes. Age and sex were included as covariates. CNV metrics (amplitude and area for iCNV, oCNV, and tCNV) were analyzed separately using LMMs. Group was specified as a between-subject fixed effect, and scalp electrode group (frontal, fronto-central, central) was specified as a within-subject fixed effect. The group-by-scalp-region interaction was tested. Participant-specific random intercepts were included, and age and sex were adjusted for. Fixed effects were evaluated using Type III tests. The Satterthwaite approximation was used to obtain degrees of freedom and *p*-values for F tests, consistent with commonly used inferential procedures for linear mixed-effects models (60). When interactions were significant, simple-effects analyses were performed. *Post hoc* pairwise comparisons were based on estimated marginal means and adjusted for multiple comparisons within each outcome using the Holm method. Model assumptions were assessed using Q–Q plots and residual diagnostics. If warranted, log transformation of the dependent variable or robust standard errors were used as sensitivity checks.

To test cross-system associations between peripheral pulsation features and central preparatory slow potentials, prespecified central–peripheral coupling LMMs were fitted in the full cohort (HC, IP, and MAP). Peripheral metrics (BPA or BPP, with repeated measurements across the three facial angiosomes) were modeled as the dependent variables, and central-region CNV amplitude was included as a continuous predictor (with iCNV and oCNV modeled in separate models). Fixed effects included group, angiosome, and the CNV × group and CNV × angiosome interaction terms. Age and sex were included as covariates, and a participant-specific random intercept was specified. Group-specific and angiosome-specific association slopes were obtained using marginal trend estimation within the estimated marginal means framework, and relevant contrasts were adjusted using the Holm method within each outcome (61).

Several targeted patient-only sensitivity analyses were conducted to evaluate distinct sources of uncertainty in the IP–MAP comparisons and, where biologically justified, in the central–peripheral association analyses. The scope of these sensitivity analyses was defined according to the presumed source of bias. Because affective symptoms may plausibly influence both central preparatory activity and peripheral hemodynamic responses, HAMA and HAMD were additionally evaluated in the central–peripheral coupling models. In contrast, HIT-6, acute symptomatic medication use, and balanced subsampling were used primarily to assess the robustness of phase-related IP–MAP group differences, because these analyses addressed headache-related impact, medication exposure, and sampling imbalance rather than the full association structure.

First, because MAP patients may exhibit higher anxiety and depressive symptom scores, we conducted sensitivity analyses to evaluate potential confounding by mood in the patient subgroup (IP and MAP), where HAMA and HAMD were available. These included: (1) adding HAMA and HAMD as covariates to the BPA/BPP group-comparison LMMs, with the same repeated-measures structure as in the primary analyses; (2) for between-group comparisons of central-region iCNV and oCNV amplitudes, which were the CNV predictors used in the central–peripheral coupling analyses, fitting multivariable linear regression models including HAMA and HAMD while adjusting for age and sex; and (3) additionally including HAMA and HAMD in the central–peripheral coupling LMMs to assess the robustness of key interaction terms (CNV × group and CNV × angiosome) and stratified slope estimates.

Second, to evaluate whether headache-related impact influenced the IP–MAP comparisons, we performed additional patient-only sensitivity analyses by including HIT-6 as a covariate. For peripheral iPPG metrics, BPA and BPP were analyzed using repeated-measures linear mixed-effects models with fixed effects for group, facial angiosome, and their interaction, a participant-specific random intercept, and adjustment for age, sex, and HIT-6. For CNV metrics, iCNV, oCNV, and tCNV amplitude and area were analyzed using repeated-measures linear mixed-effects models with fixed effects for group, scalp region, and their interaction, a participant-specific random intercept, and adjustment for age, sex, and HIT-6. These analyses were intended to evaluate whether the main IP–MAP findings were materially altered after accounting for headache-related impact.

Third, to further evaluate whether the unequal sample sizes between the IP and MAP groups influenced the phase-related findings, we performed exploratory balanced-subsample sensitivity analyses for key IP–MAP comparisons. In each of 1,000 iterations, 18 IP participants were randomly selected without replacement to match the MAP sample size, while all MAP participants were retained. MAP − IP contrasts were then re-estimated using analogous repeated-measures linear mixed-effects models with participant-specific random intercepts. These analyses were performed for peripheral iPPG metrics (BPA and BPP across the three facial angiosomes) and CNV metrics (iCNV, oCNV, and tCNV amplitude and area across the three scalp regions). Because these exploratory analyses were designed to evaluate directional stability rather than to serve as confirmatory replacements for the primary covariate-adjusted analyses, results were summarized using the median balanced estimate, the empirical 2.5–97.5% interval, and the proportion of iterations showing the same effect direction.

Fourth, to assess the potential influence of acute symptomatic medication use, we performed additional exploratory patient-only sensitivity analyses using available medication-use information. Medication exposure was coded as a binary variable indicating recorded acute symptomatic medication use versus no recorded acute symptomatic medication use. NSAIDs and, when present, other acute analgesic or triptan-related terms were coded as acute symptomatic medication use, whereas *β*-blockers and other preventive or non-acute medications were not coded as acute symptomatic medication use. For peripheral iPPG metrics, BPA and BPP were analyzed using repeated-measures LMMs with group, facial angiosome, and their interaction as fixed effects, participant-specific random intercepts, and adjustment for age, sex, and acute symptomatic medication use. For CNV metrics, iCNV, oCNV, and tCNV amplitude and area were analyzed using analogous repeated-measures LMMs with group, scalp region, and their interaction as fixed effects, participant-specific random intercepts, and adjustment for age, sex, and acute symptomatic medication use. These analyses were considered exploratory because detailed medication timing, dosage, and medication subclass information were not consistently available and because the MAP sample size was limited.

Exploratory ROC analyses were conducted to evaluate the discriminative performance of single predictors for two binary tasks: (1) migraine (IP + MAP) versus healthy controls (HC), and (2) MAP versus IP within patients. For unadjusted analyses, ROC curves were constructed separately for each single predictor, and AUCs with 95% confidence intervals were estimated using the DeLong method (62). Optimal cut-off values were determined by maximizing the Youden index (63), and the corresponding sensitivity and specificity were reported. For covariate-adjusted ROC analyses, multivariable logistic regression models were fitted with the candidate predictor as the independent variable and age and sex as covariates. Adjusted ROC curves were generated using model-predicted probabilities, and AUCs with DeLong 95% confidence intervals were calculated. Given the number of candidate predictors and the limited MAP sample size, ROC results were interpreted as within-cohort exploratory estimates of discriminative effect size and uncertainty rather than definitive diagnostic thresholds. All tests were two-sided, and *p* < 0.05 was considered statistically significant. EEG preprocessing and CNV extraction were performed in MATLAB R2024b using EEGLAB (v2022.1). Statistical analyses were conducted in R (v4.3.1).

## 3 Results

### 3.1 Demographic and clinical characteristics

A total of 150 participants were included, comprising 72 healthy controls (HC), 60 interictal patients (IP), and 18 patients assessed during a migraine attack (MAP). The three groups did not differ significantly in age, body mass index (BMI), sex distribution, or educational attainment, indicating overall comparability of baseline demographic characteristics (age: *F* = 0.45, *p* = 0.641; BMI: *F* = 0.31, *p* = 0.735; female proportion: χ^2^ = 0.05, *p* = 0.974; education: χ^2^ = 0.84, *p* = 0.991) (Table 1). Within the patient cohort (IP vs. MAP), the distribution of disease duration categories did not reach statistical significance (χ^2^ = 5.65, *p* = 0.062). Monthly headache days (MHD) and pain intensity rated by VAS were also comparable between IP and MAP (MHD: Z = −0.14, *p* = 0.896; VAS: Z = 0.22, *p* = 0.823). In contrast, HIT-6 total scores were higher in MAP than in IP (Z = −2.67, *p* = 0.007), suggesting greater headache-related disability during attacks. MAP patients also showed higher anxiety and depressive symptom scores, as assessed by HAMA and HAMD (HAMA: Z = −2.10, *p* = 0.031; HAMD: Z = −3.75, *p* < 0.001). Sleep quality measured by PSQI did not differ between groups (Z = −0.46, *p* = 0.643) (Table 1).

### 3.2 Bilateral pulsation amplitude asymmetry

**Figure 2:**
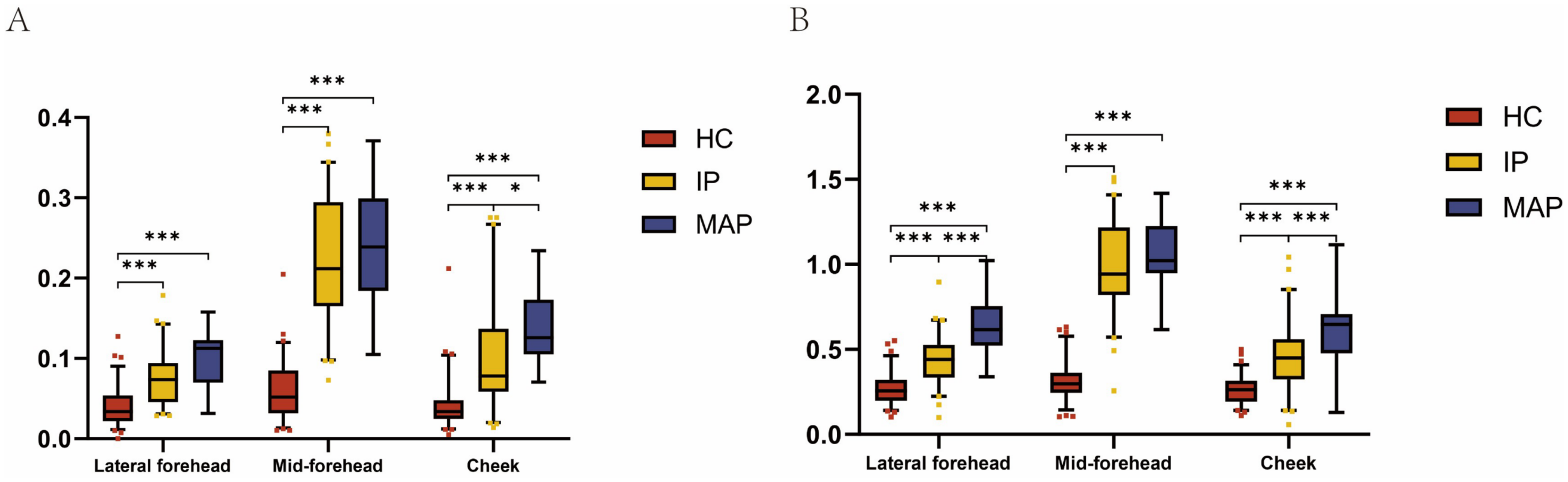
Bilateral pulsation amplitude asymmetry (BPA) and phase difference (BPP) across facial angiosomes in HC, IP, and MAP. **(A)** Bilateral pulsation amplitude asymmetry (BPA), defined as the absolute inter-side difference in pulsation amplitude. **(B)** Bilateral pulsation phase difference (BPP), defined as the absolute inter-side phase difference. HC, healthy controls (*n* = 72). IP, interictal phase (*n* = 60). MAP, migraine attack phase (*n* = 18). Lateral forehead, mid-forehead, and cheek denote the three facial angiosome groups. Boxplots show the median (center line) and interquartile range (box), with whiskers extending to 1.5 × IQR. Group differences were tested using linear mixed-effects models with fixed effects for group, angiosome, and their interaction, and a participant-specific random intercept. *Post hoc* pairwise comparisons were based on estimated marginal means and adjusted using the Holm method. Asterisks indicate within-angiosome pairwise group differences: *p < 0.05, **p < 0.01, ***p < 0.001.

Compared with healthy controls, migraine participants showed higher BPA, defined as the absolute inter-side difference in pulsation amplitude, across the three facial angiosomes (lateral forehead, mid-forehead, and cheek) (Figure 2A). Linear mixed-effects modeling with participant-specific random intercepts demonstrated significant main effects of group and angiosome, as well as a significant group-by-angiosome interaction (Group: *F*(2,147) = 169.66, *p* < 0.001; Angiosome: *F*(2,294) = 144.58, p < 0.001; Group × Angiosome: *F*(4,294) = 38.76, p < 0.001). Given the significant interaction, simple-effects analyses were performed within each angiosome using pairwise comparisons of estimated marginal means with Holm adjustment. BPA was higher in both IP and MAP than in HC across the lateral forehead, mid-forehead, and cheek (all adjusted *p* < 0.001). In addition, BPA in the cheek angiosome was higher in MAP than in IP (IP: 0.078 [0.060, 0.134] vs. MAP: 0.126 [0.111, 0.168], adjusted *p* = 0.018), whereas differences between IP and MAP were not statistically significant in the lateral forehead or mid-forehead angiosomes (adjusted *p* = 0.055 and *p* = 0.248, respectively). Overall, BPA was elevated in migraine compared with controls, and the only significant difference between MAP and IP was observed in the cheek angiosome.

### 3.3 Bilateral pulsation phase difference

Compared with healthy controls, migraine participants showed higher BPP, defined as the absolute inter-side phase difference, across the three facial angiosomes, indicating increased inter-side phase asynchrony (Figure 2B). Linear mixed-effects modeling with participant-specific random intercepts demonstrated significant main effects of group and angiosome, as well as a significant group-by-angiosome interaction (Group: *F*(2,147) = 175.07, *p* < 0.001; Angiosome: *F*(2,294) = 268.01, *p* < 0.001; Group × Angiosome: *F*(4,294) = 97.55, *p* < 0.001). Given the significant interaction, simple-effects analyses were performed within each angiosome using pairwise comparisons of estimated marginal means with Holm adjustment. BPP was higher in both IP and MAP than in HC across the lateral forehead, mid-forehead, and cheek (all adjusted *p* < 0.001). Differences between MAP and IP were angiosome-specific. MAP showed higher BPP than IP in the lateral forehead and cheek angiosomes (both adjusted *p* < 0.001), whereas no significant difference was observed in the mid-forehead angiosome (adjusted *p* = 0.221). The mid-forehead angiosome showed the highest overall BPP levels across groups, whereas the separation between MAP and IP was most evident in the lateral forehead and cheek angiosomes. Overall, BPP was elevated in migraine compared with controls, and MAP–IP differences were confined to the lateral forehead and cheek angiosomes.

### 3.4 Differences in CNV metrics

**Figure 3:**
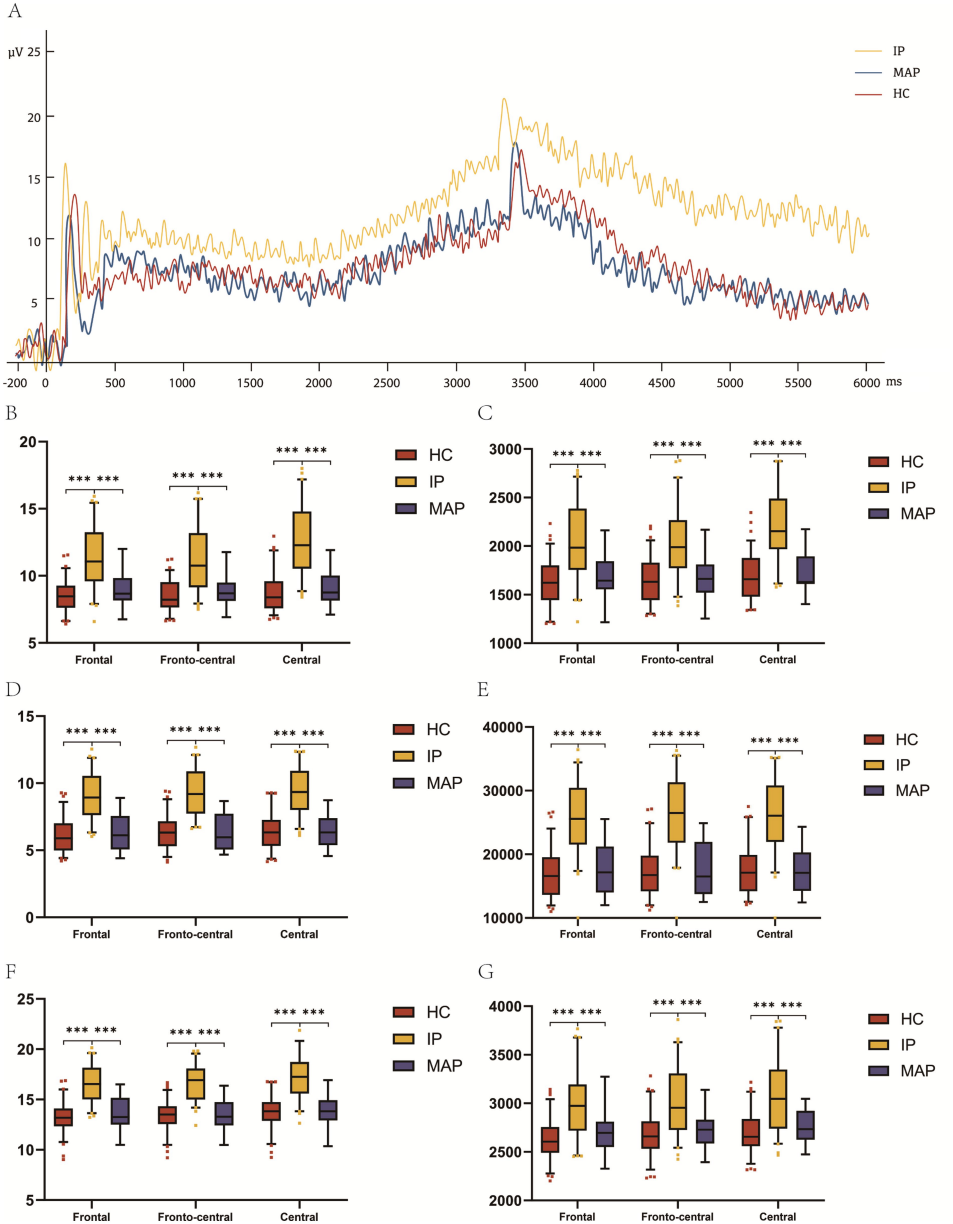
Representative CNV waveform profiles and group comparisons of CNV metrics across scalp regions. **(A)** Representative CNV waveform profiles illustrating the temporal morphology of the CNV response in healthy controls (HC), interictal-phase migraine patients (IP), and migraine-attack-phase patients (MAP). This panel is provided as a descriptive visualization of CNV temporal dynamics and was not used for group-level statistical inference. **(B)** iCNV amplitude. **(C)** iCNV area. **(D)** oCNV amplitude. **(E)** oCNV area. **(F)** tCNV amplitude. **(G)** tCNV area. HC, healthy controls (*n* = 72). IP, interictal phase (*n* = 60). MAP, migraine attack phase (*n* = 18). Frontal, fronto-central, and central denote the three scalp regions. Boxplots show the median (center line) and interquartile range (box), with whiskers extending to 1.5 × IQR. Group differences were tested using linear mixed-effects models with fixed effects for group, scalp region, and their interaction, and a participant-specific random intercept. Post hoc pairwise comparisons were based on estimated marginal means and adjusted using the Holm method. Asterisks indicate within-region pairwise group differences: *p < 0.05, **p < 0.01, ***p < 0.001. CNV values are shown after sign inversion, such that larger positive values indicate greater negative CNV amplitude or area.

Compared with healthy controls, CNV metrics across the frontal, fronto-central, and central scalp regions showed marked state-related differences in migraine (Figure 3). Representative CNV waveform profiles are shown to illustrate the temporal morphology of the CNV response across groups (Figure 3A). This waveform panel was included as a descriptive visualization, whereas group-level statistical inference was based on the predefined CNV amplitude and area metrics shown in Figures 3B–G. Linear mixed-effects models with fixed effects for group, scalp region, and their interaction, and a participant-specific random intercept, indicated significant main effects of group for all six CNV outcomes (iCNV amplitude, iCNV area, oCNV amplitude, oCNV area, tCNV amplitude, and tCNV area, all *p* < 0.001), suggesting robust between-state differences in preparatory slow potentials. Main effects of scalp region were significant for iCNV amplitude, iCNV area, oCNV amplitude, tCNV amplitude, and tCNV area (all *p* < 0.001), whereas the main effect of scalp region for oCNV area did not reach significance (*p* = 0.073). This pattern indicates region-dependent distribution of CNV magnitude overall, with comparatively less regional differentiation for oCNV area. Group-by-scalp-region interactions were significant only for iCNV amplitude and iCNV area (iCNV amplitude: *F*(4,294) = 7.90, *p* < 0.001; iCNV area: F(4,294) = 5.34, p < 0.001), indicating that group differences in iCNV depended on scalp region (Figures 3B,C). In simple-effects analyses within each scalp region using Holm-adjusted pairwise comparisons of estimated marginal means, iCNV amplitude and area were higher in IP than in both HC and MAP across all three scalp regions (all adjusted *p* < 0.001), whereas HC and MAP did not differ (all adjusted *p* > 0.05). The separation was most pronounced in the central region. For oCNV and tCNV outcomes, group-by-scalp-region interactions were not significant (all *p* > 0.05), indicating consistent group-difference patterns across scalp regions. In overall group comparisons with Holm adjustment, IP showed higher oCNV and tCNV metrics (both amplitude and area) than HC and MAP (all adjusted *p* < 0.001), whereas HC and MAP did not differ (all adjusted *p* > 0.05) (Figures 3D–G). Collectively, CNV measures were higher during the interictal phase, while values during attacks were comparable to those in healthy controls, consistent with state-dependent modulation of preparatory activity.

### 3.5 Central–peripheral association analyses

After adjustment for age and sex and accounting for repeated measurements across the three facial angiosome groups, linear mixed-effects models demonstrated significant associations between central-region CNV amplitude and peripheral pulsation metrics. Four prespecified coupling models were evaluated, including BPA–iCNV, BPA–oCNV, BPP–iCNV, and BPP–oCNV, with BPA or BPP as the dependent variable and iCNV or oCNV amplitude as the continuous predictor. Across all four models, the CNV-by-angiosome interaction was significant (all *p* < 0.001), indicating that the strength of the CNV–pulsation association differed across the lateral forehead, mid-forehead, and cheek angiosomes. The CNV-by-group interaction was also significant in each model (all *p* < 0.05), suggesting that the CNV–pulsation association varied across clinical groups (HC, IP, and MAP).

**Figure 4:**
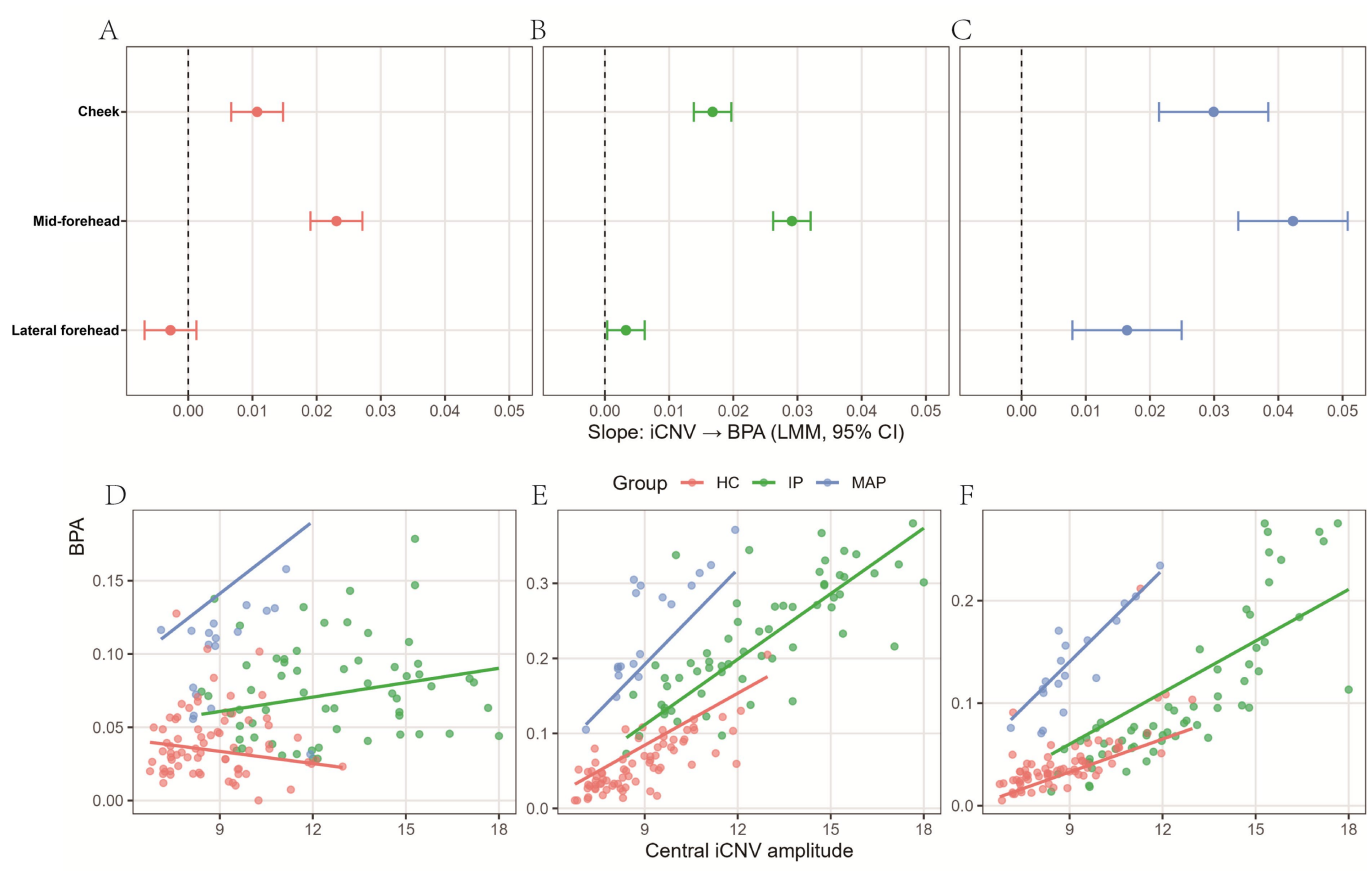
Central–peripheral coupling between iCNV amplitude and BPA across clinical groups and facial angiosomes. **(A–C)** Within-group model-estimated slopes (points) and 95% confidence intervals (horizontal lines) for the iCNV–BPA association in HC **(A)**, IP **(B)**, and MAP **(C)**. The dashed vertical line indicates the null effect (slope = 0). Each point corresponds to one predefined facial angiosome vascular territory, including the lateral forehead, mid-forehead, and cheek. These three angiosomes correspond to the territory-based facial vascular regions defined in Figure 1B and the Methods section. **(D–F)** Scatterplots of participant-level observations with model-fitted trend lines shown separately for the lateral forehead **(D)**, mid-forehead **(E)**, and cheek **(F)** angiosomes. Each dot represents one participant’s value for the corresponding angiosome. Colors indicate clinical groups: HC, healthy controls; IP, interictal phase; MAP, migraine attack phase. All slopes and fitted lines were derived from linear mixed-effects models with a participant-specific random intercept to account for within-participant correlation across angiosomes, with adjustment for age and sex. Central iCNV amplitude is shown after sign inversion, such that larger values indicate greater negative iCNV amplitude.

In the prespecified primary coupling model with BPA as the dependent variable and central-region iCNV amplitude as the continuous predictor (Figure 4), the CNV-by-group interaction was significant (*F* = 10.22, *p* < 0.001), indicating that the iCNV–BPA association differed across clinical groups. The CNV-by-angiosome interaction was also significant (*F* = 127.72, *p* < 0.001), suggesting angiosome-dependent variation in association strength. Marginal slope estimates showed a graded increase in the iCNV-to-BPA association from HC to IP and MAP: HC, 0.0104 (95% CI 0.0068–0.0139); IP, 0.0164 (95% CI 0.0141–0.0187); and MAP, 0.0295 (95% CI 0.0212–0.0379). Pairwise comparisons indicated that the MAP slope was higher than both IP and HC (Holm-adjusted *p* < 0.01 for both), and the IP slope was higher than HC (Holm-adjusted *p* < 0.001). In angiosome-stratified analyses, slopes were largest in the mid-forehead angiosome across all groups (HC: 0.0231; IP: 0.0291; MAP: 0.0423; all *p* < 0.001), followed by the cheek angiosome (HC: 0.0107; IP: 0.0168; MAP: 0.0299; all *p* < 0.001). Associations were weaker in the lateral forehead angiosome, with a non-significant slope in HC, whereas a significant positive association remained evident in MAP. Overall, the iCNV–BPA association was stronger in migraine than in controls and was most pronounced in MAP, with the largest effects observed in the mid-forehead angiosome.

In the BPA–oCNV model, we similarly observed significant CNV-by-group and CNV-by-angiosome interactions (*F* = 13.34, *p* < 0.001 and *F* = 115.64, *p* < 0.001, respectively), indicating both state dependence and angiosome dependence of the coupling pattern. Group-specific slope estimates showed the same direction as the primary BPA–iCNV analysis, with larger slopes in migraine than in controls (HC: 0.0113; IP: 0.0226; MAP: 0.0303). For BPP, both the oCNV- and iCNV-based models demonstrated significant CNV-by-group interactions (BPP–oCNV: *F* = 6.83, *p* = 0.001; BPP–iCNV: *F* = 3.39, *p* = 0.035), along with significant CNV-by-angiosome interactions (BPP–oCNV: *F* = 99.93, *p* < 0.001; BPP–iCNV: *F* = 107.21, *p* < 0.001). Across models, coupling slopes were generally larger in the mid-forehead and cheek angiosomes, whereas associations were weaker in the lateral forehead angiosome. Given the relatively small MAP sample size, slope estimates in MAP showed wider confidence intervals, and some between-group contrasts did not reach statistical significance. Detailed results are provided in Supplementary Figures 1, 2 and the Supplementary Table 1.

### 3.6 Sensitivity analyses

We performed a series of targeted patient-only sensitivity analyses to evaluate whether the main IP–MAP findings were robust to potential mood-related confounding, headache-related impact, unequal phase-specific sample sizes, and recorded acute symptomatic medication use.

Because anxiety and depressive symptom scores were higher in MAP than in IP, we performed prespecified sensitivity analyses within the patient subgroup (IP and MAP combined) to evaluate potential confounding by mood symptoms. After adjustment for HAMA and HAMD, BPA still showed a significant group main effect (*F* = 4.23, *p* = 0.043), whereas the group-by-angiosome interaction was not significant (*p* = 0.680). In prespecified angiosome contrasts, BPA remained higher in MAP than in IP in the cheek angiosome, although the effect was marginal (MAP − IP = 0.036, *p* = 0.048). BPP also showed a significant group main effect (*F* = 11.61, *p* = 0.001), and the group-by-angiosome interaction approached significance (*F* = 2.99, *p* = 0.053). Angiosome-specific contrasts indicated higher BPP in MAP than in IP in the lateral forehead (*p* < 0.001) and cheek (*p* = 0.004) angiosomes. In these models, neither HAMA nor HAMD was statistically significant. For CNV, after adjustment for HAMA and HAMD, central-region iCNV and oCNV amplitudes remained lower in MAP than in IP (iCNV: *β* = −3.38, *p* < 0.001; oCNV: β = −2.88, *p* < 0.001), suggesting that the reduction of CNV amplitudes during attacks is unlikely to be explained solely by mood symptom differences. When HAMA and HAMD were additionally included in the central–peripheral coupling models, the CNV-by-angiosome interaction remained significant across all models (all *p* < 0.001), indicating robust angiosome dependence of the coupling pattern. A significant CNV-by-group interaction persisted only in the BPA–iCNV model (*p* = 0.010), whereas group-related slope differences were not retained in the remaining coupling models. Overall, the main conclusions were broadly robust after adjustment for mood symptoms (see Supplementary Table 2).

Because HIT-6 scores were higher in MAP than in IP, we further performed HIT-6-adjusted patient-only sensitivity analyses. For peripheral iPPG metrics, MAP–IP differences were generally directionally preserved after adjustment for age, sex, and HIT-6 (see Supplementary Table 3). For BPA, adjusted MAP − IP estimates remained positive across all angiosomes but were attenuated and did not remain statistically significant after Holm correction (lateral forehead: *β* = 0.022, Holm-adjusted *p* = 0.399; mid-forehead: *β* = 0.011, Holm-adjusted *p* = 0.495; cheek: *β* = 0.027, Holm-adjusted *p* = 0.307). For BPP, MAP remained significantly higher than IP in the lateral forehead and cheek angiosomes after Holm correction (lateral forehead: *β* = 0.194, Holm-adjusted *p* = 0.003; cheek: *β* = 0.146, Holm-adjusted *p* = 0.025), whereas the mid-forehead contrast remained non-significant (*β* = 0.044, Holm-adjusted *p* = 0.442). For CNV metrics, the state-dependent differences were robust after HIT-6 adjustment: MAP showed lower sign-inverted iCNV, oCNV, and tCNV amplitude and area than IP across all scalp regions, and all corresponding contrasts remained significant after Holm correction (all Holm-adjusted *p* ≤ 0.004; see Supplementary Table 4). These findings suggest that the main CNV findings and key BPP differences were not solely attributable to differences in headache-related impact, whereas BPA-related phase differences should be interpreted more cautiously.

To evaluate whether the unequal IP and MAP sample sizes influenced the phase-related findings, we performed exploratory balanced-subsample sensitivity analyses. In each of 1,000 iterations, IP participants were randomly subsampled to match the MAP sample size, and key IP–MAP comparisons were re-estimated using analogous repeated-measures linear mixed-effects models. For peripheral iPPG metrics, the directions of MAP–IP differences were generally preserved (see Supplementary Table 5). MAP showed higher BPA than IP in 100.0% of iterations for the lateral forehead, 83.1% for the mid-forehead, and 98.9% for the cheek. For BPP, MAP showed higher values than IP in 100.0% of iterations for the lateral forehead, 86.9% for the mid-forehead, and 100.0% for the cheek. The empirical intervals were entirely above zero for the lateral forehead and cheek regions but crossed zero for the mid-forehead, indicating weaker stability in this region. For sign-inverted CNV metrics, the directional findings were highly stable: MAP showed lower iCNV, oCNV, and tCNV amplitude and area than IP across all scalp regions in 100.0% of iterations, with empirical intervals consistently below zero (see Supplementary Table 6). These results suggest that the main phase-related findings were not solely driven by the larger IP sample, although MAP-related estimates should still be interpreted cautiously because of the limited MAP sample size.

We also performed exploratory medication-adjusted sensitivity analyses using available binary information on acute symptomatic medication use. Acute symptomatic medication use was recorded in 28 of 60 IP participants (46.7%) and 11 of 18 MAP participants (61.1%). After adjustment for age, sex, and acute symptomatic medication use, the directions of MAP–IP differences in peripheral iPPG metrics were generally preserved (see Supplementary Table 7). For BPA, adjusted MAP − IP estimates remained positive across all angiosomes but did not remain statistically significant after Holm correction (lateral forehead: *β* = 0.026, Holm-adjusted *p* = 0.247; mid-forehead: *β* = 0.015, Holm-adjusted *p* = 0.351; cheek: *β* = 0.031, Holm-adjusted *p* = 0.174). For BPP, MAP remained significantly higher than IP in the lateral forehead and cheek angiosomes after Holm correction (lateral forehead: *β* = 0.214, Holm-adjusted *p* < 0.001; cheek: *β* = 0.165, Holm-adjusted *p* = 0.007), whereas the mid-forehead contrast remained non-significant (*β* = 0.064, Holm-adjusted *p* = 0.257). For sign-inverted CNV metrics, medication adjustment did not materially alter the state-dependent differences: MAP showed lower iCNV, oCNV, and tCNV amplitude and area than IP across all scalp regions, and all corresponding contrasts remained significant after Holm correction (all Holm-adjusted *p* ≤ 0.002; see Supplementary Table 8). These findings suggest that the major CNV findings and key BPP differences were not solely attributable to recorded acute symptomatic medication use, whereas BPA-related phase differences should be interpreted more cautiously.

Taken together, these sensitivity analyses supported the robustness of the CNV-related state effects and the key BPP findings, while indicating that BPA-related phase differences were less stable after adjustment for headache-related impact and acute symptomatic medication use.

### 3.7 Exploratory ROC analyses

**Figure 5:**
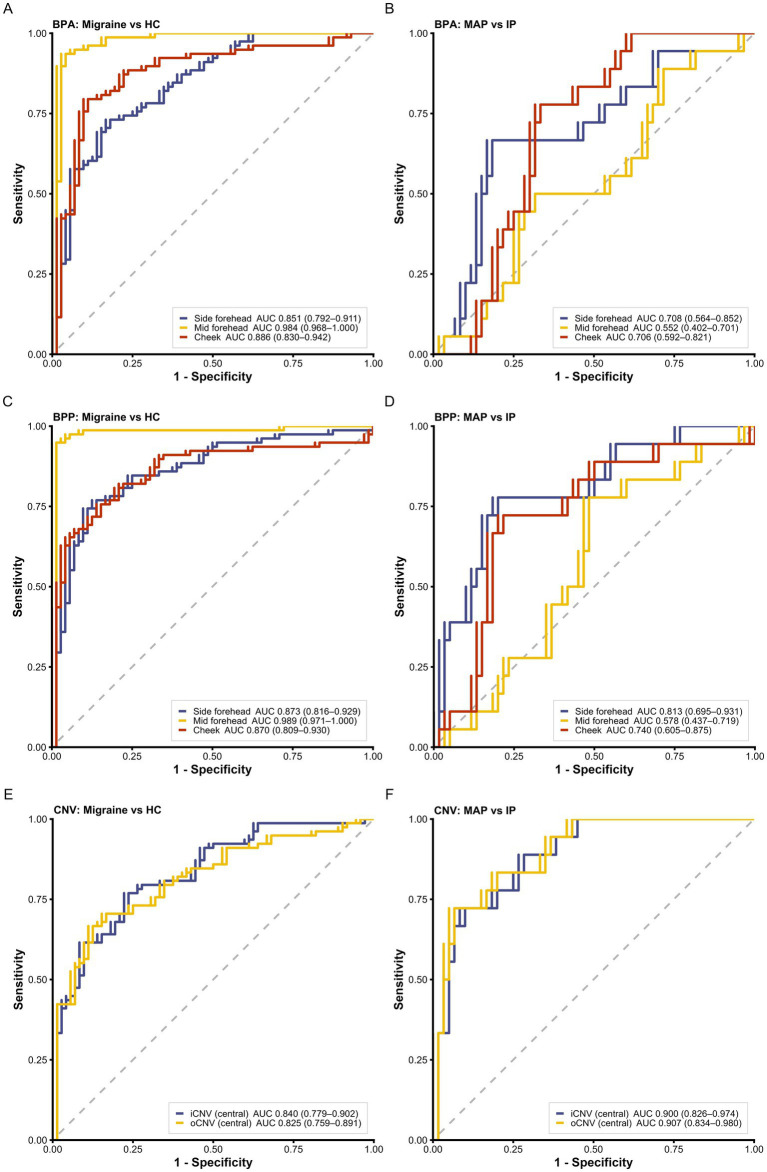
Exploratory within-cohort ROC curves for peripheral pulsation metrics and central CNV amplitudes. **(A–D)** BPA and BPP from the lateral forehead, mid-forehead, and cheek angiosomes for migraine (IP + MAP) versus HC and for MAP versus IP within patients. **(E,F)** Central-region iCNV and oCNV amplitudes for the same two classification tasks. AUCs with 95% confidence intervals were estimated using the DeLong method. The gray dashed diagonal indicates chance-level discrimination. The positive class was migraine for panels **A**, **C**, and **E** and MAP for panels **B**, **D**, and **F**. These ROC analyses describe within-cohort separability and should not be interpreted as a deployable diagnostic model or a definitive clinical threshold.

In unadjusted within-cohort exploratory ROC analyses, mid-forehead pulsation metrics showed high distributional separability for migraine (IP and MAP combined) versus healthy controls (HC) (BPA: AUC ≈ 0.984; BPP: AUC ≈ 0.989) (Figure 5). Lateral forehead and cheek metrics also demonstrated good to excellent separability (AUC ≈ 0.851–0.873). Central-region CNV amplitudes showed moderate to good separability for migraine versus HC (iCNV: AUC ≈ 0.845; oCNV: AUC ≈ 0.832). For the within-patient task of MAP versus IP, CNV metrics yielded the highest separability (iCNV: AUC ≈ 0.902; oCNV: AUC ≈ 0.913). In contrast, BPA and BPP showed reduced separability for MAP versus IP but remained in the moderate range across angiosomes (AUC ≈ 0.621–0.832). After adjustment for age and sex, the overall AUC ranking pattern remained consistent with the unadjusted analyses, suggesting that demographic differences were unlikely to fully account for the observed within-cohort separability pattern (Supplementary Figure 3). These ROC analyses are exploratory and intended to describe single-predictor, within-cohort separability rather than to establish generalizable diagnostic performance or deployable thresholds.

## 4 Discussion

In this study, we combined facial iPPG-based assessment of superficial pulsation features with a classic S1–S2 paradigm to derive CNV. This joint peripheral–central approach enabled profiling of state-related migraine phenotypes from both peripheral hemodynamics and central preparatory slow potentials. We report three main findings. First, compared with healthy controls, migraine participants showed increased BPA and BPP across the lateral forehead, mid-forehead, and cheek angiosomes, indicating greater inter-side asymmetry and phase asynchrony in superficial vascular pulsation. Second, CNV metrics exhibited a clear state-dependent profile. Interictal iCNV, oCNV, and tCNV measures, including both amplitude and area, were consistently higher than those observed in healthy controls and during attacks, whereas CNV values during the migraine attack phase were generally comparable to control levels. Third, central–peripheral association analyses revealed significant positive relationships between CNV amplitudes and peripheral pulsation metrics. These association patterns showed robust angiosome-dependent modulation, with group-related slope differences most evident in iCNV-related models, particularly for BPA–iCNV coupling. Targeted sensitivity analyses supported the robustness of the CNV-related state effects and key BPP findings after adjustment for mood symptoms, headache-related impact, acute symptomatic medication use, and after balanced-subsample evaluation of the unequal IP and MAP sample sizes. In contrast, BPA-related phase differences were less stable after adjustment for headache-related impact and medication exposure and should therefore be interpreted more cautiously. Exploratory ROC analyses suggested within-cohort separability for selected peripheral and CNV metrics, but clinical utility requires replication in larger samples and independent external cohorts. Overall, our findings provide an interpretable link between facial iPPG-derived pulsation features and CNV-indexed preparatory activity and offer quantitative evidence supporting state-dependent neurovascular dysregulation in migraine.

iPPG offers a non-contact and low-barrier approach to rapidly capture cardiac-related pulsation signals from superficial microcirculation, providing a feasible pathway for quantifying peripheral phenotypes of neurovascular regulatory disorders such as migraine in real-world settings (64, 65). In this study, we used the trans-angiosome imaging photoplethysmography (TaiPPG) framework to quantify inter-side pulsation amplitude asymmetry and phase asynchrony within distinct facial angiosomes and observed consistent directional differences across migraine clinical states, supporting the potential utility of these peripheral measures as candidate signals for screening and stratification. At the same time, iPPG remains sensitive to measurement noise. Short recordings can be influenced by residual head and facial micro-movements, transient autonomic fluctuations, and changes in ambient illumination (66). To mitigate these effects, we implemented multiple controls during acquisition and processing, including head stabilization with a chin rest, orthogonal polarization to reduce specular reflection, exclusion of periorbital and perioral regions prone to micro-movement artifacts, and lock-in amplification to enhance cardiac-related components and improve signal-to-noise ratio. Importantly, our primary peripheral endpoints were constructed as inter-side differences within paired angiosomes. This design can partially suppress global perturbations that affect both sides similarly, such as common-mode illumination changes or synchronized motion. Because random measurement error typically attenuates effect estimates toward the null, the observed group differences and central–peripheral associations may represent conservative estimates. Given that this work represents an early application of iPPG in migraine, future studies should evaluate robustness and generalizability in larger samples using longer recordings or repeated acquisitions with averaging. Incorporating objective motion quantification, such as landmark displacement, video quality metrics, or motion-regression terms, may further strengthen reliability under real-world conditions.

Peripheral findings indicated that migraine was associated with greater BPA and BPP across facial superficial vessels, suggesting altered modulation of cardiac-related perfusion dynamics in the craniofacial vascular bed. This phenotype is compatible with the trigeminovascular framework. Activation of trigeminal afferents can promote the release of vasoactive neuropeptides, including CGRP, VIP, and substance P, which may contribute to vasodilation, increased vascular permeability, and neurogenic inflammation. These peripheral vascular responses could amplify nociceptive input and interact with central pain processing (15, 67). Within this interpretive framework, higher BPA reflects greater inter-side asymmetry in pulsation amplitude modulation, whereas higher BPP reflects reduced inter-side temporal alignment of pulsations, consistent with desynchronization of rhythmic vascular responses. The more pronounced MAP–IP differences observed in specific angiosomes are consistent with spatial heterogeneity of peripheral vascular dysregulation. In our data, the MAP–IP separation was confined to the cheek angiosome for BPA and to the lateral forehead and cheek angiosomes for BPP. Such heterogeneity may be influenced by regional differences in arterial supply and in the contribution of trigeminal–autonomic reflex pathways, although these mechanisms were not directly tested here (68, 69). Because CGRP-targeted therapies reduce attack burden in a subset of patients, BPA and BPP may provide quantitative peripheral phenotypes for future studies evaluating state-dependent vascular responses related to neuropeptide pathways (70, 71). At the same time, our metrics were based on absolute inter-side differences. This design reduces sensitivity to common-mode perturbations but does not preserve directional laterality. Future work integrating headache laterality, pain intensity, and vasoactive biomarkers such as CGRP may help clarify correspondence between peripheral pulsation features and clinical phenotypes and strengthen mechanistic interpretation.

Migraine is not only characterized by recurrent pain attacks but is also associated with fluctuating impairments in attention, working memory, and executive control (72–74). As a preparatory slow potential, CNV reflects a continuous control process spanning anticipatory attention and response preparation. Stage-specific components, including iCNV, oCNV, and tCNV, are commonly interpreted as indexing early alerting, sustained resource allocation, and terminal preparation, respectively. Consistent with prior reports describing interictal enhancement with relative attenuation during attacks, we observed higher iCNV, oCNV, and tCNV measures, including both amplitude and area, across multiple scalp regions in IP compared with both HC and MAP, whereas differences between MAP and HC were comparatively small (36). This pattern supports a state-dependent modulation of preparatory neural activity in migraine. Elevated interictal CNV may reflect heightened predictive processing of warning cues and increased readiness to respond, which is compatible with concepts of central sensitization and altered habituation or information-processing strategies reported in migraine (32, 39, 75, 76). In contrast, during attacks, ongoing pain burden, altered arousal, and reallocation of attentional resources may contribute to an attenuation of preparatory activity, yielding CNV levels that appear closer to those of healthy controls at the group level. Future studies could increase trial numbers, quantify reliability using split-half and test–retest approaches, and integrate source localization or time–frequency phenotypes, such as preparatory theta and delta activity, to more directly test the proposed sequence of interictal gain enhancement and attack-related attenuation and to improve interpretability and reproducibility.

A further contribution of this work is that we placed peripheral vascular pulsation features and central preparatory slow potentials within a single statistical framework to directly test cross-system associations between central state and peripheral vascular rhythms. In the strict sense, neurovascular coupling most commonly refers to the correspondence between neural activity and local cerebral blood flow responses. Altered neurovascular coupling has been linked to network-level dysfunction and symptom fluctuations in migraine (77). Recent work combining arterial spin labeling measures of cerebral blood flow with resting-state fMRI indices of local activity, such as regional homogeneity, suggests reduced intracerebral neurovascular coupling in migraine and potential associations with disease burden or chronicity (78). Multi-delay ASL studies have further reported phase- and subtype-dependent dynamics in cerebral perfusion, highlighting state dependence and spatiotemporal heterogeneity of flow regulation (79–83). Against this background, our findings provide a potentially translatable peripheral window. Importantly, the “coupling” examined here does not refer to classical intracerebral neurovascular coupling. Instead, it refers to subject-level statistical associations between CNV-indexed central preparatory activity and iPPG-derived peripheral microcirculatory pulsation asymmetry and asynchrony metrics, namely BPA and BPP. Mechanistic interpretation should therefore emphasize cross-system co-modulation by shared regulatory pathways, rather than a direct hemodynamic matching of local cerebral blood flow to neural activity.

In our models, CNV, which reflects anticipatory attention, motivational engagement, and motor preparation and is commonly linked to frontoparietal control and motor-preparatory networks, was significantly associated with facial microcirculatory pulsation metrics (BPA and BPP). These associations were angiosome dependent, suggesting that fluctuations in central preparatory gain may co-vary with dysregulated craniofacial peripheral vascular rhythms in migraine. Because clinical state (IP versus MAP) can shift group means in both CNV and iPPG-derived metrics, we explicitly modeled group and angiosome effects and examined within-group slopes in the mixed-effects framework. We therefore interpret the findings as evidence of state-related cross-system co-variation rather than an ecological correlation driven solely by between-group mean differences. A causal interpretation that CNV directly produces peripheral asymmetry or asynchrony is not warranted. A more plausible account is that both central preparatory activity and peripheral vascular rhythms are jointly modulated by shared state-dependent pathways, including central autonomic control networks and trigeminal–autonomic reflex mechanisms. Under conditions of elevated or more variable preparatory gain, peripheral vascular smooth muscle tone and rhythmic synchrony may be more prone to inter-side and territory-level mismatch, resulting in stronger CNV–pulsation coupling. This mechanistic explanation remains hypothetical and requires validation in studies incorporating concurrent autonomic measurements or experimental perturbations. Moreover, the observed angiosome dependence further indicates that peripheral vascular territories do not respond uniformly to central regulatory signals, which may relate to differences in local vascular architecture, innervation, or measurement sensitivity. Importantly, the present analyses are observational. The coupling pattern reflects state-related association rather than causation.

In addition, heart rate variability (HRV) and beat-to-beat heart rate or interbeat-interval recordings may be particularly informative in future CNV–iPPG studies, because CNV paradigms are often accompanied by anticipatory cardiac deceleration during the S1–S2 interval, a component of the orienting response that has been linked to enhanced stimulus processing and motor preparatory control (84–87). Such concurrent autonomic recordings could help determine the extent to which CNV–iPPG associations are mediated or modulated by autonomic regulation during anticipatory preparation. Future work using longitudinal follow-up, multimodal autonomic monitoring such as HRV, electrodermal activity, or pupillometry, and pre–post comparisons around CGRP-targeted treatments may help determine whether this cross-system coupling can serve as a candidate marker of migraine state or treatment response.

To provide an intuitive visualization of within-cohort distributional separability and to complement group-effect estimates, we performed exploratory ROC analyses. These ROC analyses were intended for descriptive evaluation only and do not constitute evidence for a deployable diagnostic model or definitive clinical thresholds. Peripheral pulsation metrics showed relatively high separability for migraine (IP + MAP) versus healthy controls, with the highest AUCs observed for the mid-forehead angiosome. AUC values approaching 1.0 are not uncommon in single-predictor separability assessments conducted within the same cohort using a shared acquisition platform and preprocessing pipeline, particularly when the two groups exhibit limited distributional overlap on a given feature. In the present study, BPA and BPP were constructed as inter-side difference metrics within paired angiosomes. This design can partially suppress common-mode perturbations such as illumination and camera-gain fluctuations and may concentrate between-group differences along the asymmetry and asynchrony dimensions, thereby inflating within-cohort separability. However, such within-cohort separability should not be equated with generalizable performance across centers, devices, or more heterogeneous real-world conditions. Central-region CNV amplitudes also showed moderate separability for migraine versus healthy controls, although overall discrimination was lower than that of the mid-forehead peripheral metrics. The relatively stronger mid-forehead performance may reflect both biological and technical factors. Biologically, fluctuations in trigeminovascular and autonomic tone in migraine may perturb peripheral vasomotor regulation and rhythmic synchrony, amplifying inter-side asymmetry and phase mismatch. Technically, the mid-forehead territory may be more uniform in mask consistency, skin texture, and illumination geometry, which could reduce random variability in short recordings, although this explanation was not directly tested.

For the within-patient task of MAP versus IP, central CNV measures outperformed peripheral pulsation metrics, consistent with the pronounced state dependence observed for CNV, with higher preparatory activity during IP and attenuation during MAP. This pattern suggests that fluctuations in central preparatory and attentional resource allocation may more directly index attack-related neurophysiological state differences than peripheral vascular rhythms, at least in the present experimental context. In covariate-adjusted models including age and sex, the overall ranking pattern of AUCs was consistent with the unadjusted analyses, suggesting that demographic differences were unlikely to fully account for the observed separability patterns within this cohort. These ROC analyses were exploratory and conducted within a single center cohort. Given the small MAP sample size and the absence of external validation, AUCs and cut-offs may be optimistic and should be interpreted as preliminary.

Despite integrating territory-based facial iPPG features with CNV-indexed preparatory activity within a unified analytical framework, several limitations should be acknowledged. First, phase-specific sampling and clinical-state differences should be considered when interpreting the IP–MAP comparisons. The MAP sample size was relatively small (*n* = 18) and smaller than the IP sample, which may have reduced statistical power, widened confidence intervals, and increased the sensitivity of MAP-related estimates to individual observations. Linear mixed-effects models can accommodate unequal group sizes, but unequal sampling can still affect the precision of phase-specific estimates. To address this issue, we performed exploratory balanced-subsample sensitivity analyses by repeatedly subsampling the IP group to match the MAP sample size. These analyses suggested that the direction of peripheral iPPG contrasts was generally preserved, particularly for BPA and BPP in the lateral forehead and cheek regions, whereas mid-forehead effects were less stable. CNV-related IP–MAP differences were highly stable in direction across amplitude and area metrics and across scalp regions. In addition, HIT-6 scores were higher in MAP than in IP, which is clinically expected because MAP participants were assessed during symptomatic attacks. HIT-6 may therefore partly reflect the attack-related clinical state rather than a fully independent confounder. HIT-6-adjusted sensitivity analyses showed that CNV-related differences remained robust and that BPP differences in the lateral forehead and cheek angiosomes were preserved, whereas BPA contrasts were attenuated. Taken together, findings involving MAP, especially BPA-related peripheral effects, should be interpreted cautiously and require confirmation in larger, prospectively recruited cohorts with more balanced phase-specific sample sizes.

Second, although headache phase classification was verified bidirectionally using symptom status at acquisition and headache diaries that recorded attack onset and offset, pain intensity, acute medication use, and prodromal and postdromal symptoms, misclassification cannot be fully excluded. Mild prodromal or postdromal symptoms and inter-individual differences in symptom recognition may have blurred phase boundaries. To mitigate this risk, we applied a conservative interictal definition, requiring no headache at acquisition, more than 24 h since the end of the previous attack, and more than 24 h before the onset of the subsequent attack, with no recorded prodromal or postdromal symptoms within the prespecified window. Future studies could extend the headache-free window to 48–72 h, implement prospective high-frequency monitoring, and conduct stratified analyses according to prodromal, ictal, postdromal, and interictal status.

Third, in real-world outpatient settings, some participants, particularly those assessed during the attack phase, may have used acute symptomatic medications around acquisition, most commonly NSAIDs. Such medications could influence peripheral hemodynamic signals and central preparatory activity through analgesic effects, modulation of inflammatory mediators and vasoactive pathways, and altered autonomic responses. In response to this concern, we performed exploratory medication-adjusted sensitivity analyses using available binary medication-use information. These analyses indicated that the major CNV differences remained robust and that BPP differences in the lateral forehead and cheek angiosomes were preserved, whereas BPA contrasts were directionally preserved but not statistically significant after adjustment. Nevertheless, detailed information regarding exact medication timing, dosage, and medication subclass was limited, and the MAP sample size was small. Therefore, residual medication-related confounding cannot be excluded. Future work should standardize acquisition windows and systematically capture medication timing, dose, drug class, and treatment response to better evaluate medication-related and autonomic confounding.

Fourth, our peripheral pulsation metrics were defined using absolute inter-side differences to quantify the magnitude of asymmetry (BPA) and phase asynchrony (BPP). This construction reduces sensitivity to inter-individual facial geometry and common-mode noise but does not preserve directional laterality. Because headache laterality was not systematically recorded and aligned with the iPPG acquisition, we were unable to test whether peripheral asymmetry corresponded to the side of pain. Future studies could incorporate headache side and pain intensity at acquisition to derive signed laterality metrics and evaluate their concordance with clinical lateralization, which would strengthen mechanistic interpretation and clinical relevance.

Fifth, measurement-related limitations should also be noted. Behavioral measures were not treated as primary outcomes in this study. The CNV paradigm was designed as a simple reaction task, for which accuracy is expected to be near ceiling and between-group differences, if present, often manifest as modest shifts in reaction time. Given our focus on electrophysiological markers of preparatory state and their association with peripheral pulsation features, behavioral indices were not prioritized as primary endpoints. Nevertheless, pain state could influence CNV through changes in attention, arousal, or motor execution. We applied standardized preprocessing and artifact control procedures to minimize systematic differences in signal quality across groups. Future work should consider reporting retained trial counts and evaluating the impact of trial numbers and signal-to-noise differences on CNV estimates. In addition, both iPPG-derived pulsation features and CNV measures are susceptible to measurement noise and state fluctuations. Although we implemented multiple procedures to reduce artifacts and enhance signal quality, additional work is needed to quantify reliability using longer recordings and repeated acquisitions with averaging. Future studies should also assess metric stability and strengthen robustness and reproducibility.

Sixth, this study used a cross-sectional design for state comparisons and association analyses. The observed central–peripheral coupling therefore reflects state-related co-variation and should not be interpreted as evidence of causality. Longitudinal follow-up, tracking natural state transitions, and pre–post comparisons around CGRP-targeted therapy or neuromodulation interventions will be important to determine whether peripheral pulsation features and their coupling with CNV change dynamically with symptoms and have predictive value. Future studies incorporating concurrent autonomic recordings, such as HRV, electrodermal activity, or pupillometry, may also help clarify whether CNV–iPPG coupling is mediated or modulated by autonomic regulation during anticipatory preparation.

Finally, generalizability and model validation remain limited. Anxiety and depression scores were not collected in healthy controls, which prevented adjustment for mood symptoms in migraine versus control comparisons. Although we conducted mood-adjusted sensitivity analyses within patients, residual confounding by unmeasured psychological factors cannot be fully excluded at the cohort level. ROC analyses were exploratory and conducted within a single-center cohort. Given the limited MAP sample size, AUCs and Youden-based cut-offs may be optimistic and influenced by spectrum and selection bias. These findings should therefore be interpreted as preliminary within-cohort separability estimates and require validation in independent external cohorts. Future studies should also evaluate generalizability using prespecified validation frameworks, including internal resampling or external validation, calibration assessment, and decision-curve analysis. Although we controlled multiple comparisons in the primary analyses using Holm adjustment within outcomes, different correction strategies can influence the interpretation of borderline findings. Accordingly, results near conventional significance thresholds should be interpreted with emphasis on effect direction and consistency and confirmed in larger samples.

Despite these limitations, our study provides converging evidence that facial iPPG-derived pulsation asymmetry and asynchrony, together with CNV-indexed preparatory activity, show state-dependent alterations in migraine. The observed angiosome-dependent central–peripheral associations support an integrative framework linking peripheral hemodynamics with central electrophysiology for future longitudinal and interventional research. Further validation in larger cohorts and independent external samples is warranted to clarify robustness and potential translational utility.

## 5 Conclusion

We quantified facial pulsation asymmetry and phase asynchrony across migraine states using iPPG and assessed preparatory slow potentials indexed by CNV. Migraine was associated with increased peripheral pulsation asymmetry and phase differences across facial angiosomes, while CNV showed a state-dependent profile with higher measures during the interictal phase and attenuation during attacks. Central–peripheral analyses further demonstrated angiosome-dependent positive associations between CNV amplitudes and peripheral pulsation features, supporting state-dependent central–peripheral co-variation in migraine. Exploratory ROC analyses suggested within-cohort separability for selected peripheral and CNV metrics, but replication in larger and independent cohorts is needed to establish robustness and translational value.

## Data Availability

The data supporting the findings of this study are not publicly available due to institutional and ethical restrictions, but may be obtained from the corresponding author upon reasonable request and with appropriate data use agreements.
