# Supplementary material for: State-dependent facial pulsation asymmetry and phase asynchrony measured by imaging photoplethysmography and their coupling with contingent negative variation in migraine
Source: Front Neurol. 2026 Jun 4;17:1818649. doi: 10.3389/fneur.2026.1818649 (PMC13275365; doi:10.3389/fneur.2026.1818649)
Supplement: Supplementary file 1 [file Supplementary_file_1.docx]

Supplementary Material

# Supplementary Figures and Tables

## Supplementary Figures


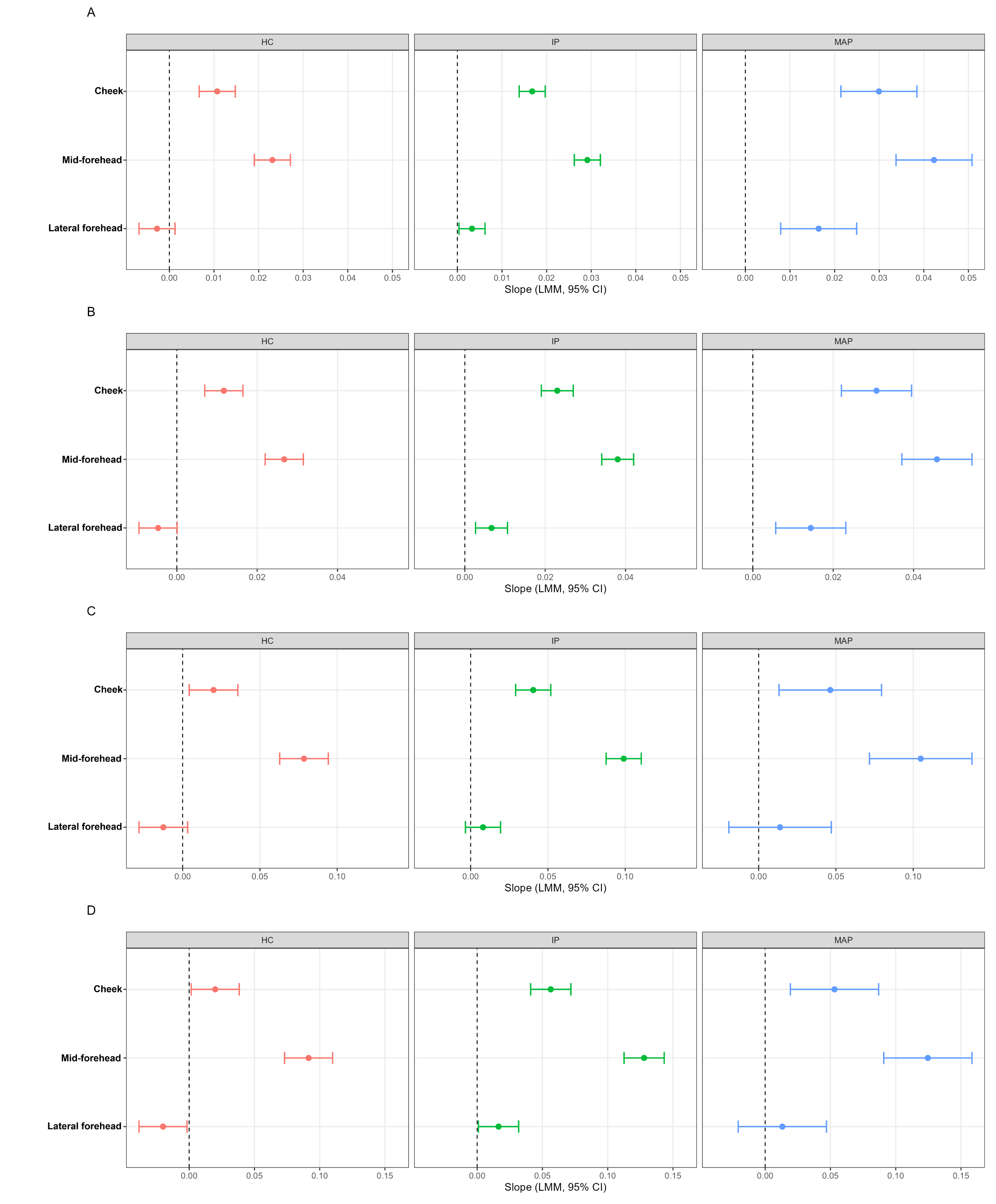


**Supplementary Figure 1.** **Forest plots of model-estimated slopes for the association between CNV amplitude and facial pulsation lateralization across models.**

(A) BPA–iCNV; (B) BPA–oCNV; (C) BPP–iCNV; and (D) BPP–oCNV. Points indicate the fixed-effect slope estimates (β) from linear mixed-effects models (LMMs), and horizontal lines represent 95% confidence intervals. The vertical dashed line denotes the null effect (β = 0). Within each panel, results are stratified by clinical group—HC (healthy controls), IP (interictal phase), and MAP (migraine attack phase)—and further reported for each facial angiosome (lateral forehead, mid-forehead, and cheek). All models adjusted for age and sex and included a participant-specific random intercept to account for within-participant correlation arising from repeated measurements across angiosomes. BPA, bilateral pulsation amplitude asymmetry; BPP, bilateral pulsation phase difference; iCNV/oCNV, central CNV amplitude metrics (expressed as the absolute value of the negative-going component; larger values indicate greater negative amplitude).


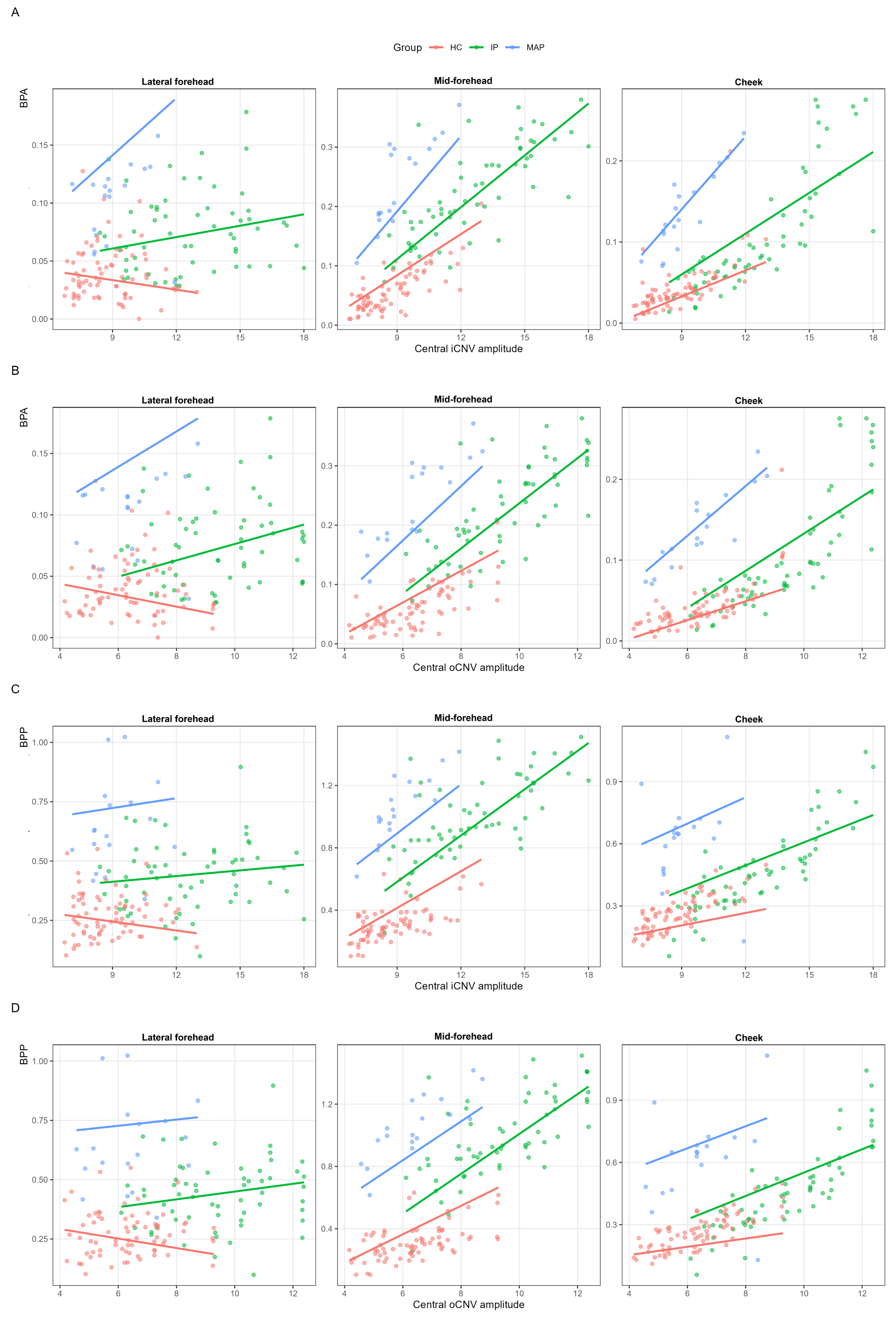


**Supplementary Figure 2. Distributions of raw observations and LMM marginal fitted trends across models.**

(A) BPA–iCNV; (B) BPA–oCNV; (C) BPP–iCNV; and (D) BPP–oCNV. Scatter points represent participant-level observations for each facial angiosome (lateral forehead, mid-forehead, and cheek), with colors indicating clinical group (HC, IP, and MAP). Solid lines denote the marginal fitted trends from linear mixed-effects models (LMMs), derived from fixed-effect predictions after adjustment for age and sex and accounting for within-participant correlation due to repeated measurements across angiosomes via a participant-specific random intercept. Fitted lines are displayed only within the observed CNV range for each group × angiosome stratum to avoid extrapolation beyond the available data. Abbreviations are as in Supplementary Figure 1.


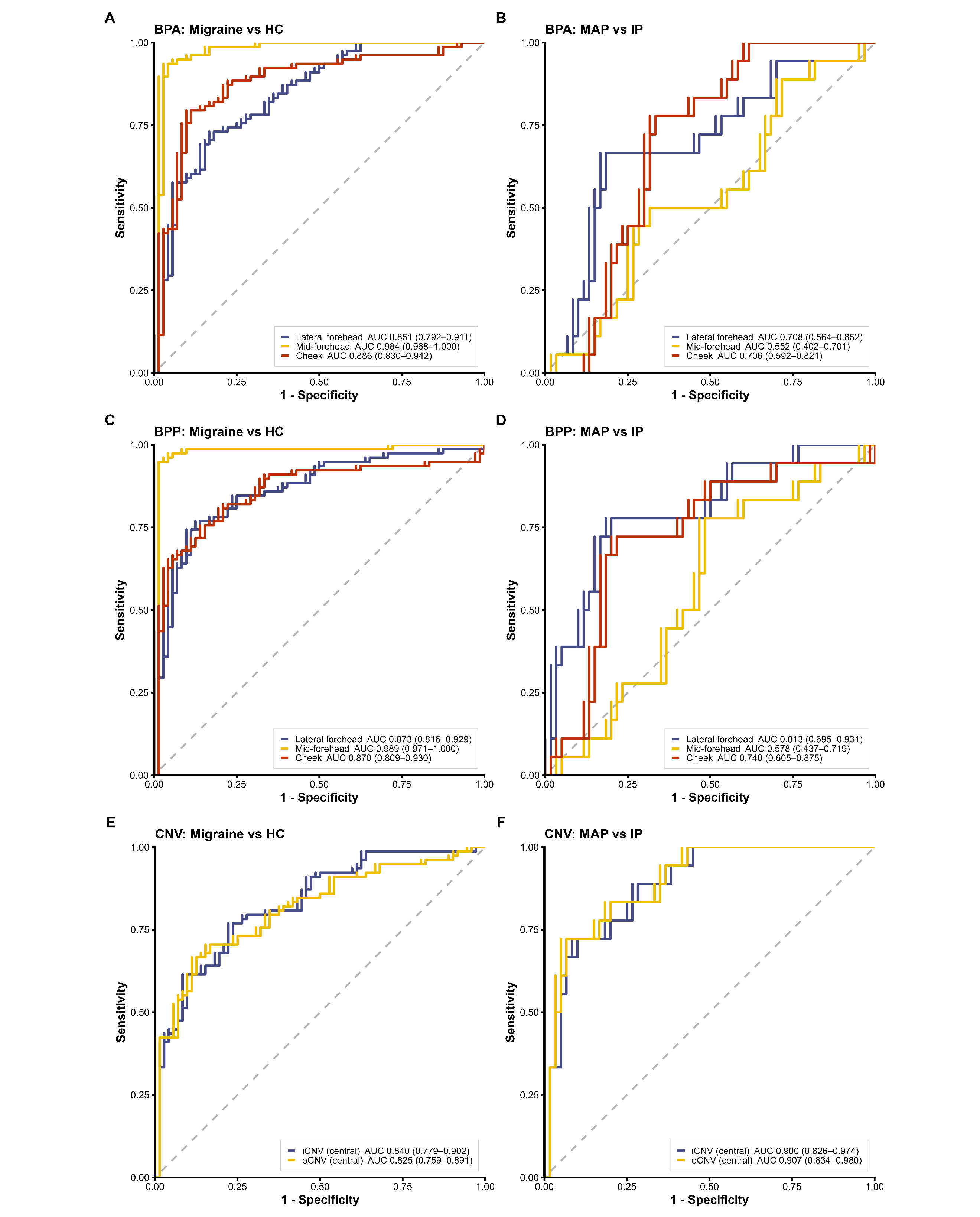


**Supplementary Figure 3.** **Exploratory age- and sex-adjusted within-cohort ROC curves for peripheral pulsation metrics and central CNV amplitudes.**

ROC curves were derived from multivariable logistic regression models including one candidate marker at a time with simultaneous adjustment for age and sex; model-predicted probabilities were used to generate covariate-adjusted ROC curves. (A–D) BPA and BPP from the lateral forehead, mid-forehead, and cheek angiosomes for migraine (IP + MAP) versus HC and for MAP versus IP within patients. (E–F) Central-region iCNV and oCNV amplitudes for the same two classification tasks. AUCs with 95% confidence intervals were estimated using the DeLong method. The gray dashed diagonal indicates chance-level discrimination. The positive class was migraine for panels A, C, and E and MAP for panels B, D, and F. These ROC analyses describe within-cohort separability and should not be interpreted as a deployable diagnostic model or a definitive clinical threshold.

## Supplementary Tables

## Supplementary Table 1. Key effects and slope estimates for the four central–peripheral coupling models.

| **Model** | **CNV×Group** | **CNV×Angiosome** | **β-HC (95%CI)** | **β-IP (95%CI)** | **β-MAP (95%CI)** | **Δβ IP–HC (Holm p)** | **Δβ MAP–HC (Holm p)** | **Δβ MAP–IP (Holm p)** | **N (observations)** |
| --- | --- | --- | --- | --- | --- | --- | --- | --- | --- |
| **BPA-iCNV** | F=10.22; P=4.56e-05 | F=127.72; P=1.17e-44 | 0.0104 [0.0068, 0.0139] | 0.0164 [0.0141, 0.0187] | 0.0295 [0.0212, 0.0379] | 0.0060; 0.006 | 0.0192; 0.0001 | 0.0132; 0.006 | 450 |
| **BPA-oCNV** | F=13.34; P=2.36e-06 | F=115.64; P=2.98e-41 | 0.0113 [0.0071, 0.0154] | 0.0226 [0.0194, 0.0258] | 0.0303 [0.0219, 0.0387] | 0.0113; 8.31e-05 | 0.0191; 0.0002 | 0.0078; 0.0903 | 450 |
| **BPP-iCNV** | F=3.39; P=0.0347 | F=107.21; P=8.37e-39 | 0.0287 [0.0147, 0.0427] | 0.0492 [0.0404, 0.0580] | 0.0550 [0.0227, 0.0874] | 0.0205; 0.0437 | 0.0264; 0.2826 | 0.0059; 0.7302 | 450 |
| **BPP-oCNV** | F=6.83; P=0.0012 | F=99.93; P=1.23e-36 | 0.0305 [0.0145, 0.0464] | 0.0669 [0.0545, 0.0793] | 0.0636 [0.0311, 0.0962] | 0.0364; 0.0013 | 0.0332; 0.146 | -0.0032; 0.8545 | 450 |

**Note.** β denotes the marginal CNV-to-peripheral association slope estimated from linear mixed-effects models (LMMs) (units: BPA/BPP per 1 µV CNV). Slopes were summarized as estimated marginal trends over facial angiosomes under models including the CNV × angiosome interaction. Δβ denotes between-group differences in slopes; P values are Holm-adjusted. All models adjusted for age and sex and included a participant-specific random intercept to account for within-participant correlation due to repeated measurements across the three angiosomes. HC, healthy controls; IP, interictal phase; MAP, migraine attack phase. BPA, bilateral pulsation amplitude asymmetry. BPP, bilateral pulsation phase difference. iCNV/oCNV, central-region CNV amplitude metrics.

## Supplementary Table 2. Sensitivity analyses adjusting for emotional factors (HAMA and HAMD) in the patient subgroup

1. **Group comparisons of pulsation metrics (BPA/BPP) in patients (IP vs MAP)**

| **Outcome** | **Effect (Type III)** | **NumDF** | **DenDF** | **F** | **p** |
| --- | --- | --- | --- | --- | --- |
| **BPA** | Group (IP vs MAP) | 1 | 78.00 | 4.23 | 0.043 |
|  | Angiosome (lateral/mid-forehead/cheek) | 2 | 156.00 | 134.28 | <0.001 |
|  | Age | 1 | 78.00 | 2.02 | 0.160 |
|  | Sex | 1 | 78.00 | 0.12 | 0.732 |
|  | HAMA | 1 | 78.00 | 0.08 | 0.779 |
|  | HAMD | 1 | 78.00 | 0.45 | 0.505 |
|  | Group × Angiosome | 2 | 156.00 | 0.39 | 0.680 |
| **BPP** | Group (IP vs MAP) | 1 | 78.00 | 11.61 | 0.001 |
|  | Angiosome (lateral/mid-forehead/cheek) | 2 | 156.00 | 158.97 | <0.001 |
|  | Age | 1 | 78.00 | 1.94 | 0.168 |
|  | Sex | 1 | 78.00 | 0.20 | 0.656 |
|  | HAMA | 1 | 78.00 | 0.06 | 0.812 |
|  | HAMD | 1 | 78.00 | 0.24 | 0.627 |
|  | Group × Angiosome | 2 | 156.00 | 2.99 | 0.053 |

1. **Planned contrasts (EMMs): IP − MAP, stratified by angiosome (Negative estimates indicate higher values in MAP than in IP)**

| **Outcome** | **Angiosome** | **Estimate (IP − MAP)** | **SE** | **df** | **t** | **p** |
| --- | --- | --- | --- | --- | --- | --- |
| **BPA** | Lateral forehead | −0.0303 | 0.0181 | 178.24 | −1.67 | 0.097 |
|  | Mid-forehead | −0.0202 | 0.0181 | 178.24 | −1.11 | 0.268 |
|  | Cheek | −0.0362 | 0.0181 | 178.24 | −1.99 | 0.048 |
| **BPP** | Lateral forehead | −0.2218 | 0.0600 | 184.89 | −3.70 | <0.001 |
|  | Mid-forehead | −0.0719 | 0.0600 | 184.89 | −1.20 | 0.232 |
|  | Cheek | −0.1731 | 0.0600 | 184.89 | −2.89 | 0.004 |

1. **Group comparisons of central CNV amplitudes (iCNV/oCNV) in patients (IP vs MAP)**

| **Outcome** | **Term** | **β** | **SE** | **t** | **p** |
| --- | --- | --- | --- | --- | --- |
| **iCNV (central)** | Intercept | 10.680 | 1.923 | 5.55 | <0.001 |
|  | Group: MAP (vs IP) | −3.379 | 0.704 | −4.80 | <0.001 |
|  | Age | 0.037 | 0.027 | 1.37 | 0.174 |
|  | Sex: female (vs male) | 0.432 | 0.565 | 0.77 | 0.446 |
|  | HAMA | 0.018 | 0.146 | 0.12 | 0.902 |
|  | HAMD | −0.023 | 0.125 | −0.18 | 0.856 |
| **oCNV (central)** | Intercept | 8.449 | 1.432 | 5.90 | <0.001 |
|  | Group: MAP (vs IP) | −2.877 | 0.524 | −5.49 | <0.001 |
|  | Age | 0.023 | 0.020 | 1.16 | 0.249 |
|  | Sex: female (vs male) | 0.285 | 0.421 | 0.68 | 0.500 |
|  | HAMA | −0.007 | 0.109 | −0.06 | 0.949 |
|  | HAMD | −0.032 | 0.093 | −0.34 | 0.733 |

1. **Central–peripheral coupling models in patients with HAMA/HAMD adjustment (Type III tests)**

| **Model** | **Effect (Type III)** | **NumDF** | **DenDF** | **F** | **p** |
| --- | --- | --- | --- | --- | --- |
| **BPA–iCNV (central)** | CNV (main effect) | 1 | 78.00 | 87.68 | <0.001 |
|  | Group (IP vs MAP) | 1 | 78.00 | 0.51 | 0.478 |
|  | Angiosome | 2 | 156.00 | 13.17 | <0.001 |
|  | Age | 1 | 78.00 | 0.26 | 0.612 |
|  | Sex | 1 | 78.00 | 0.64 | 0.426 |
|  | HAMA | 1 | 78.00 | 0.00 | 0.958 |
|  | HAMD | 1 | 78.00 | 0.49 | 0.488 |
|  | CNV × Group | 1 | 78.00 | 6.93 | 0.010 |
|  | CNV × Angiosome | 2 | 156.00 | 30.79 | <0.001 |
| **BPA–oCNV (central)** | CNV (main effect) | 1 | 234.00 | 108.07 | <0.001 |
|  | Group (IP vs MAP) | 1 | 234.00 | 1.74 | 0.188 |
|  | Angiosome | 2 | 234.00 | 10.70 | <0.001 |
|  | Age | 1 | 234.00 | 0.73 | 0.394 |
|  | Sex | 1 | 234.00 | 0.28 | 0.595 |
|  | HAMA | 1 | 234.00 | 0.09 | 0.759 |
|  | HAMD | 1 | 234.00 | 0.35 | 0.555 |
|  | CNV × Group | 1 | 234.00 | 2.00 | 0.158 |
|  | CNV × Angiosome | 2 | 234.00 | 26.29 | <0.001 |
| **BPP–iCNV (central)** | CNV (main effect) | 1 | 78.00 | 26.05 | <0.001 |
|  | Group (IP vs MAP) | 1 | 78.00 | 1.83 | 0.180 |
|  | Angiosome | 2 | 156.00 | 11.66 | <0.001 |
|  | Age | 1 | 78.00 | 0.26 | 0.614 |
|  | Sex | 1 | 78.00 | 0.05 | 0.821 |
|  | HAMA | 1 | 78.00 | 0.27 | 0.603 |
|  | HAMD | 1 | 78.00 | 0.23 | 0.632 |
|  | CNV × Group | 1 | 78.00 | 0.09 | 0.760 |
|  | CNV × Angiosome | 2 | 156.00 | 27.00 | <0.001 |
| **BPP–oCNV (central)** | CNV (main effect) | 1 | 78.00 | 38.03 | <0.001 |
|  | Group (IP vs MAP) | 1 | 78.00 | 5.70 | 0.019 |
|  | Angiosome | 2 | 156.00 | 11.26 | <0.001 |
|  | Age | 1 | 78.00 | 0.53 | 0.470 |
|  | Sex | 1 | 78.00 | 0.01 | 0.922 |
|  | HAMA | 1 | 78.00 | 0.06 | 0.804 |
|  | HAMD | 1 | 78.00 | 0.12 | 0.735 |
|  | CNV × Group | 1 | 78.00 | 0.01 | 0.922 |
|  | CNV × Angiosome | 2 | 156.00 | 25.71 | <0.001 |

1. **Stratified slopes (estimated marginal trends): CNV → pulsation lateralization within each Group × Angiosome.**

| **Model** | **Group** | **Angiosome** | **Slope β** | **95% CI** | **p** |
| --- | --- | --- | --- | --- | --- |
| **BPA–iCNV** | IP | Lateral forehead | 0.0055 | 0.0016–0.0093 | 0.006 |
|  | MAP | Lateral forehead | 0.0184 | 0.0081–0.0286 | <0.001 |
|  | IP | Mid-forehead | 0.0230 | 0.0191–0.0269 | <0.001 |
|  | MAP | Mid-forehead | 0.0359 | 0.0256–0.0462 | <0.001 |
|  | IP | Cheek | 0.0210 | 0.0171–0.0248 | <0.001 |
|  | MAP | Cheek | 0.0338 | 0.0236–0.0441 | <0.001 |
| **BPA–oCNV** | IP | Lateral forehead | 0.0094 | 0.0041–0.0146 | <0.001 |
|  | MAP | Lateral forehead | 0.0166 | 0.0060–0.0273 | 0.002 |
|  | IP | Mid-forehead | 0.0307 | 0.0255–0.0359 | <0.001 |
|  | MAP | Mid-forehead | 0.0380 | 0.0274–0.0486 | <0.001 |
|  | IP | Cheek | 0.0279 | 0.0226–0.0331 | <0.001 |
|  | MAP | Cheek | 0.0351 | 0.0245–0.0458 | <0.001 |
| **BPP–iCNV** | IP | Lateral forehead | 0.0143 | −0.0006–0.0292 | 0.061 |
|  | MAP | Lateral forehead | 0.0205 | −0.0222–0.0632 | 0.342 |
|  | IP | Mid-forehead | 0.0755 | 0.0606–0.0904 | <0.001 |
|  | MAP | Mid-forehead | 0.0818 | 0.0391–0.1245 | <0.001 |
|  | IP | Cheek | 0.0580 | 0.0431–0.0729 | <0.001 |
|  | MAP | Cheek | 0.0643 | 0.0216–0.1070 | 0.004 |
| **BPP–oCNV** | IP | Lateral forehead | 0.0226 | 0.0024–0.0428 | 0.028 |
|  | MAP | Lateral forehead | 0.0205 | −0.0235–0.0645 | 0.357 |
|  | IP | Mid-forehead | 0.0997 | 0.0795–0.1199 | <0.001 |
|  | MAP | Mid-forehead | 0.0976 | 0.0536–0.1416 | <0.001 |
|  | IP | Cheek | 0.0776 | 0.0574–0.0978 | <0.001 |
|  | MAP | Cheek | 0.0755 | 0.0315–0.1195 | <0.001 |

1. **Between-group differences in slopes (IP − MAP; negative values indicate larger slopes in MAP)**

| **Model** | **Slope difference (IP − MAP)** | **SE** | **df** | **t** | **p** |
| --- | --- | --- | --- | --- | --- |
| BPA–iCNV | −0.0129 | 0.0052 | 86.91 | −2.49 | 0.015 |
| BPA–oCNV | −0.0073 | 0.0054 | 86.02 | −1.34 | 0.182 |
| BPP–iCNV | −0.0063 | 0.0217 | 86.91 | −0.29 | 0.773 |
| BPP–oCNV | 0.0021 | 0.0228 | 86.91 | 0.09 | 0.926 |

**Supplementary Table 3. HIT-6-adjusted sensitivity analyses for IP–MAP comparisons of peripheral iPPG metrics.**

| **Metric** | **Facial angiosome** | **HIT-6-adjusted estimate (MAP − IP)** | **SE** | **p value** | **Holm-adjusted p value** | **Direction** | **Interpretation** |
| --- | --- | --- | --- | --- | --- | --- | --- |
| BPA | Lateral forehead | 0.022 | 0.017 | 0.199 | 0.399 | MAP > IP | Direction preserved; not significant after Holm adjustment |
| BPA | Mid-forehead | 0.011 | 0.017 | 0.495 | 0.495 | MAP > IP | Direction preserved; not significant after Holm adjustment |
| BPA | Cheek | 0.027 | 0.017 | 0.102 | 0.307 | MAP > IP | Direction preserved; not significant after Holm adjustment |
| BPP | Lateral forehead | 0.194 | 0.058 | <0.001 | 0.003 | MAP > IP | Direction preserved; significant after Holm adjustment |
| BPP | Mid-forehead | 0.044 | 0.058 | 0.442 | 0.442 | MAP > IP | Direction preserved; not significant after Holm adjustment |
| BPP | Cheek | 0.146 | 0.058 | 0.013 | 0.025 | MAP > IP | Direction preserved; significant after Holm adjustment |

Note. HIT-6-adjusted sensitivity analyses were performed within the patient cohort, including only IP and MAP participants. BPA and BPP were analyzed separately using repeated-measures linear mixed-effects models with group, facial angiosome, and their interaction as fixed effects, participant-specific random intercepts, and adjustment for age, sex, and HIT-6. Estimates represent MAP − IP contrasts within each facial angiosome. Positive estimates indicate higher values in MAP than in IP. Holm-adjusted p values were calculated across the three angiosome-specific MAP − IP contrasts within each metric. BPA, bilateral pulsation amplitude asymmetry; BPP, bilateral pulsation phase difference; HIT-6, 6-item Headache Impact Test; iPPG, imaging photoplethysmography; IP, interictal phase; MAP, migraine attack phase; SE, standard error.

## Supplementary Table 4. HIT-6-adjusted sensitivity analyses for IP–MAP comparisons of CNV amplitude and area metrics.

| **Outcome** | **Scalp region** | **HIT-6-adjusted estimate (MAP − IP)** | **SE** | **p value** | **Holm-adjusted p value** | **Direction** | **Interpretation** |
| --- | --- | --- | --- | --- | --- | --- | --- |
| iCNV amplitude | Frontal | −2.329 | 0.617 | <0.001 | <0.001 | MAP < IP | Direction preserved; significant after Holm adjustment |
| iCNV amplitude | Fronto-central | −2.230 | 0.617 | <0.001 | <0.001 | MAP < IP | Direction preserved; significant after Holm adjustment |
| iCNV amplitude | Central | −3.524 | 0.617 | <0.001 | <0.001 | MAP < IP | Direction preserved; significant after Holm adjustment |
| iCNV area | Frontal | −363.942 | 97.687 | <0.001 | <0.001 | MAP < IP | Direction preserved; significant after Holm adjustment |
| iCNV area | Fronto-central | −355.230 | 97.687 | <0.001 | <0.001 | MAP < IP | Direction preserved; significant after Holm adjustment |
| iCNV area | Central | −488.312 | 97.687 | <0.001 | <0.001 | MAP < IP | Direction preserved; significant after Holm adjustment |
| oCNV amplitude | Frontal | −2.838 | 0.473 | <0.001 | <0.001 | MAP < IP | Direction preserved; significant after Holm adjustment |
| oCNV amplitude | Fronto-central | −3.117 | 0.473 | <0.001 | <0.001 | MAP < IP | Direction preserved; significant after Holm adjustment |
| oCNV amplitude | Central | −3.054 | 0.473 | <0.001 | <0.001 | MAP < IP | Direction preserved; significant after Holm adjustment |
| oCNV area | Frontal | −8694.715 | 1420.823 | <0.001 | <0.001 | MAP < IP | Direction preserved; significant after Holm adjustment |
| oCNV area | Fronto-central | −9467.174 | 1420.823 | <0.001 | <0.001 | MAP < IP | Direction preserved; significant after Holm adjustment |
| oCNV area | Central | −9179.520 | 1420.823 | <0.001 | <0.001 | MAP < IP | Direction preserved; significant after Holm adjustment |
| tCNV amplitude | Frontal | −3.129 | 0.533 | <0.001 | <0.001 | MAP < IP | Direction preserved; significant after Holm adjustment |
| tCNV amplitude | Fronto-central | −3.121 | 0.533 | <0.001 | <0.001 | MAP < IP | Direction preserved; significant after Holm adjustment |
| tCNV amplitude | Central | −3.566 | 0.533 | <0.001 | <0.001 | MAP < IP | Direction preserved; significant after Holm adjustment |
| tCNV area | Frontal | −302.914 | 94.116 | 0.002 | 0.004 | MAP < IP | Direction preserved; significant after Holm adjustment |
| tCNV area | Fronto-central | −291.258 | 94.116 | 0.003 | 0.004 | MAP < IP | Direction preserved; significant after Holm adjustment |
| tCNV area | Central | −321.384 | 94.116 | <0.001 | 0.003 | MAP < IP | Direction preserved; significant after Holm adjustment |

**Note.** HIT-6-adjusted sensitivity analyses were performed within the patient cohort, including only IP and MAP participants. Each CNV outcome was analyzed separately using repeated-measures linear mixed-effects models with group, scalp region, and their interaction as fixed effects, participant-specific random intercepts, and adjustment for age, sex, and HIT-6. Estimates represent MAP − IP contrasts within each scalp region. Because CNV metrics were analyzed after sign inversion, negative estimates indicate smaller CNV magnitude in MAP than in IP. Holm-adjusted p values were calculated across the three scalp-region-specific MAP − IP contrasts within each CNV outcome. CNV, contingent negative variation; HIT-6, 6-item Headache Impact Test; iCNV, initial contingent negative variation; IP, interictal phase; MAP, migraine attack phase; oCNV, overall contingent negative variation; SE, standard error; tCNV, terminal contingent negative variation.

## Supplementary Table 5. Balanced-subsample sensitivity analyses for IP–MAP comparisons of BPA and BPP.

| **Metric** | **Region** | **Full-sample estimate (MAP − IP)** | **Full-sample p value** | **Median balanced estimate (MAP − IP)** | **Empirical 2.5%–97.5% interval** | **MAP > IP across iterations (%)** | **p < 0.05 across iterations (%)** | **Interpretation** |
| --- | --- | --- | --- | --- | --- | --- | --- | --- |
| BPA | Lateral forehead | 0.025 | 0.129 | 0.025 | 0.013 to 0.038 | 100.0 | 1.5 | Highly stable direction; empirical interval > 0 |
| BPA | Mid-forehead | 0.015 | 0.360 | 0.016 | −0.013 to 0.045 | 83.1 | 8.7 | Moderately stable direction; empirical interval crosses 0 |
| BPA | Cheek | 0.031 | 0.062 | 0.031 | 0.004 to 0.058 | 98.9 | 30.0 | Highly stable direction; empirical interval > 0 |
| BPP | Lateral forehead | 0.203 | <0.001 | 0.202 | 0.149 to 0.259 | 100.0 | 99.0 | Highly stable direction; empirical interval > 0 |
| BPP | Mid-forehead | 0.054 | 0.334 | 0.056 | −0.047 to 0.155 | 86.9 | 6.6 | Moderately stable direction; empirical interval crosses 0 |
| BPP | Cheek | 0.155 | 0.006 | 0.156 | 0.076 to 0.228 | 100.0 | 68.9 | Highly stable direction; empirical interval > 0 |

**Note.** In each of 1,000 iterations, 18 IP participants were randomly selected without replacement to match the MAP sample size, while all MAP participants were retained. MAP − IP contrasts were estimated using an analogous repeated-measures linear mixed-effects model with participant-specific random intercepts. Positive estimates indicate higher values in MAP than in IP. The analysis was intended to assess directional stability rather than to establish resampling-based statistical significance. The empirical 2.5%–97.5% interval represents the distribution of MAP − IP estimates across the 1,000 balanced iterations. Abbreviations: BPA, bilateral pulsation amplitude asymmetry; BPP, bilateral pulsation phase difference; iPPG, imaging photoplethysmography; IP, interictal phase; MAP, migraine attack phase.

## Supplementary Table 6. Balanced-subsample sensitivity analyses for IP–MAP comparisons of CNV amplitude and area metrics.

| **Outcome** | **Scalp region** | **Full-sample estimate (MAP − IP)** | **Full-sample p value** | **Median balanced estimate (MAP − IP)** | **Empirical 2.5%–97.5% interval** | **MAP < IP across iterations (%)** | **p < 0.05 across iterations (%)** | **Interpretation** |
| --- | --- | --- | --- | --- | --- | --- | --- | --- |
| iCNV amplitude | Frontal | −2.304 | 1.46 × 10⁻⁴ | −2.297 | −3.161 to −1.433 | 100.0 | 99.4 | Highly stable direction: MAP < IP |
| iCNV amplitude | Fronto-central | −2.205 | 2.70 × 10⁻⁴ | −2.225 | −3.096 to −1.366 | 100.0 | 98.6 | Highly stable direction: MAP < IP |
| iCNV amplitude | Central | −3.499 | 2.66 × 10⁻⁸ | −3.530 | −4.514 to −2.605 | 100.0 | 100.0 | Highly stable direction: MAP < IP |
| iCNV area | Frontal | −356.748 | 1.92 × 10⁻⁴ | −356.776 | −510.168 to −219.224 | 100.0 | 98.9 | Highly stable direction: MAP < IP |
| iCNV area | Fronto-central | −348.036 | 2.69 × 10⁻⁴ | −349.310 | −495.787 to −214.071 | 100.0 | 98.7 | Highly stable direction: MAP < IP |
| iCNV area | Central | −481.118 | 9.18 × 10⁻⁷ | −482.281 | −615.038 to −340.127 | 100.0 | 100.0 | Highly stable direction: MAP < IP |
| oCNV amplitude | Frontal | −2.803 | 1.29 × 10⁻⁸ | −2.799 | −3.451 to −2.156 | 100.0 | 100.0 | Highly stable direction: MAP < IP |
| oCNV amplitude | Fronto-central | −3.081 | 7.74 × 10⁻¹⁰ | −3.073 | −3.756 to −2.415 | 100.0 | 100.0 | Highly stable direction: MAP < IP |
| oCNV amplitude | Central | −3.018 | 1.47 × 10⁻⁹ | −2.995 | −3.713 to −2.311 | 100.0 | 100.0 | Highly stable direction: MAP < IP |
| oCNV area | Frontal | −8524.356 | 9.53 × 10⁻⁹ | −8512.958 | −10571.278 to −6532.114 | 100.0 | 100.0 | Highly stable direction: MAP < IP |
| oCNV area | Fronto-central | −9296.815 | 7.12 × 10⁻¹⁰ | −9293.157 | −11243.962 to −7282.869 | 100.0 | 100.0 | Highly stable direction: MAP < IP |
| oCNV area | Central | −9009.161 | 1.88 × 10⁻⁹ | −9008.631 | −11100.077 to −6926.793 | 100.0 | 100.0 | Highly stable direction: MAP < IP |
| tCNV amplitude | Frontal | −3.086 | 1.69 × 10⁻⁸ | −3.103 | −3.822 to −2.334 | 100.0 | 100.0 | Highly stable direction: MAP < IP |
| tCNV amplitude | Fronto-central | −3.077 | 1.83 × 10⁻⁸ | −3.093 | −3.750 to −2.397 | 100.0 | 100.0 | Highly stable direction: MAP < IP |
| tCNV amplitude | Central | −3.522 | 2.92 × 10⁻¹⁰ | −3.551 | −4.314 to −2.657 | 100.0 | 100.0 | Highly stable direction: MAP < IP |
| tCNV area | Frontal | −306.749 | 8.72 × 10⁻⁴ | −303.645 | −445.574 to −177.527 | 100.0 | 96.6 | Highly stable direction: MAP < IP |
| tCNV area | Fronto-central | −295.092 | 1.33 × 10⁻³ | −292.132 | −432.550 to −173.972 | 100.0 | 95.5 | Highly stable direction: MAP < IP |
| tCNV area | Central | −325.218 | 4.38 × 10⁻⁴ | −323.212 | −480.864 to −184.140 | 100.0 | 98.1 | Highly stable direction: MAP < IP |

**Note.** In each of 1,000 iterations, 18 IP participants were randomly subsampled without replacement to match the MAP sample size, while all MAP participants were retained. MAP − IP contrasts were estimated using an analogous repeated-measures linear mixed-effects model with participant-specific random intercepts. Because CNV metrics were analyzed after sign inversion, negative MAP − IP estimates indicate smaller CNV magnitude in MAP than in IP. The analysis was intended to assess directional stability rather than to replace the primary adjusted analyses. The empirical 2.5%–97.5% interval represents the distribution of MAP − IP estimates across the 1,000 balanced iterations. Abbreviations: CNV, contingent negative variation; iCNV, initial contingent negative variation; oCNV, overall contingent negative variation; tCNV, terminal contingent negative variation; IP, interictal phase; MAP, migraine attack phase.

## Supplementary Table 7. Medication-adjusted sensitivity analyses for IP–MAP comparisons of peripheral iPPG metrics.

| **Metric** | **Facial angiosome** | **Medication-adjusted estimate (MAP − IP)** | **SE** | **p value** | **Holm-adjusted p value** | **Direction** | **Interpretation** |
| --- | --- | --- | --- | --- | --- | --- | --- |
| BPA | Lateral forehead | 0.026 | 0.017 | 0.123 | 0.247 | MAP > IP | Direction preserved; not significant after Holm adjustment |
| BPA | Mid-forehead | 0.015 | 0.017 | 0.351 | 0.351 | MAP > IP | Direction preserved; not significant after Holm adjustment |
| BPA | Cheek | 0.031 | 0.017 | 0.058 | 0.174 | MAP > IP | Direction preserved; not significant after Holm adjustment |
| BPP | Lateral forehead | 0.214 | 0.056 | <0.001 | <0.001 | MAP > IP | Direction preserved; significant after Holm adjustment |
| BPP | Mid-forehead | 0.064 | 0.056 | 0.257 | 0.257 | MAP > IP | Direction preserved; not significant after Holm adjustment |
| BPP | Cheek | 0.165 | 0.056 | 0.004 | 0.007 | MAP > IP | Direction preserved; significant after Holm adjustment |

**Note.** Medication-adjusted sensitivity analyses were performed within the patient cohort, including only IP and MAP participants. Available medication-use information was coded as a binary covariate indicating recorded acute symptomatic medication use versus no recorded acute symptomatic medication use. NSAIDs and other acute analgesic or triptan-related terms were coded as acute symptomatic medication use, whereas β-blockers and other preventive or non-acute medications were not coded as acute symptomatic medication use. BPA and BPP were analyzed separately using repeated-measures linear mixed-effects models with group, facial angiosome, and their interaction as fixed effects, participant-specific random intercepts, and adjustment for age, sex, and acute symptomatic medication use. Estimates represent MAP − IP contrasts within each facial angiosome. Positive estimates indicate higher values in MAP than in IP. Holm-adjusted p values were calculated across the three angiosome-specific MAP − IP contrasts within each metric. BPA, bilateral pulsation amplitude asymmetry; BPP, bilateral pulsation phase difference; iPPG, imaging photoplethysmography; IP, interictal phase; MAP, migraine attack phase; NSAIDs, nonsteroidal anti-inflammatory drugs; SE, standard error.

## Supplementary Table 8. Medication-adjusted sensitivity analyses for IP–MAP comparisons of CNV amplitude and area metrics.

| **Outcome** | **Scalp region** | **Medication-adjusted estimate (MAP − IP)** | **SE** | **p value** | **Holm-adjusted p value** | **Direction** | **Interpretation** |
| --- | --- | --- | --- | --- | --- | --- | --- |
| iCNV amplitude | Frontal | −2.263 | 0.590 | <0.001 | <0.001 | MAP < IP | Direction preserved; significant after Holm adjustment |
| iCNV amplitude | Fronto-central | −2.164 | 0.590 | <0.001 | <0.001 | MAP < IP | Direction preserved; significant after Holm adjustment |
| iCNV amplitude | Central | −3.458 | 0.590 | <0.001 | <0.001 | MAP < IP | Direction preserved; significant after Holm adjustment |
| iCNV area | Frontal | −353.908 | 94.044 | <0.001 | <0.001 | MAP < IP | Direction preserved; significant after Holm adjustment |
| iCNV area | Fronto-central | −345.196 | 94.044 | <0.001 | <0.001 | MAP < IP | Direction preserved; significant after Holm adjustment |
| iCNV area | Central | −478.278 | 94.044 | <0.001 | <0.001 | MAP < IP | Direction preserved; significant after Holm adjustment |
| oCNV amplitude | Frontal | −2.809 | 0.454 | <0.001 | <0.001 | MAP < IP | Direction preserved; significant after Holm adjustment |
| oCNV amplitude | Fronto-central | −3.088 | 0.454 | <0.001 | <0.001 | MAP < IP | Direction preserved; significant after Holm adjustment |
| oCNV amplitude | Central | −3.025 | 0.454 | <0.001 | <0.001 | MAP < IP | Direction preserved; significant after Holm adjustment |
| oCNV area | Frontal | −8534.251 | 1366.275 | <0.001 | <0.001 | MAP < IP | Direction preserved; significant after Holm adjustment |
| oCNV area | Fronto-central | −9306.710 | 1366.275 | <0.001 | <0.001 | MAP < IP | Direction preserved; significant after Holm adjustment |
| oCNV area | Central | −9019.056 | 1366.275 | <0.001 | <0.001 | MAP < IP | Direction preserved; significant after Holm adjustment |
| tCNV amplitude | Frontal | −3.086 | 0.514 | <0.001 | <0.001 | MAP < IP | Direction preserved; significant after Holm adjustment |
| tCNV amplitude | Fronto-central | −3.077 | 0.514 | <0.001 | <0.001 | MAP < IP | Direction preserved; significant after Holm adjustment |
| tCNV amplitude | Central | −3.522 | 0.514 | <0.001 | <0.001 | MAP < IP | Direction preserved; significant after Holm adjustment |
| tCNV area | Frontal | −309.048 | 90.410 | <0.001 | 0.002 | MAP < IP | Direction preserved; significant after Holm adjustment |
| tCNV area | Fronto-central | −297.391 | 90.410 | 0.001 | 0.002 | MAP < IP | Direction preserved; significant after Holm adjustment |
| tCNV area | Central | −327.517 | 90.410 | <0.001 | 0.001 | MAP < IP | Direction preserved; significant after Holm adjustment |

**Note.** Medication-adjusted sensitivity analyses were performed within the patient cohort, including only IP and MAP participants. Available medication-use information was coded as a binary covariate indicating recorded acute symptomatic medication use versus no recorded acute symptomatic medication use. NSAIDs and other acute analgesic or triptan-related terms were coded as acute symptomatic medication use, whereas β-blockers and other preventive or non-acute medications were not coded as acute symptomatic medication use. Each CNV outcome was analyzed separately using repeated-measures linear mixed-effects models with group, scalp region, and their interaction as fixed effects, participant-specific random intercepts, and adjustment for age, sex, and acute symptomatic medication use. Estimates represent MAP − IP contrasts within each scalp region. Because CNV metrics were analyzed after sign inversion, negative estimates indicate smaller CNV magnitude in MAP than in IP. Holm-adjusted p values were calculated across the three scalp-region-specific MAP − IP contrasts within each CNV outcome. CNV, contingent negative variation; iCNV, initial contingent negative variation; IP, interictal phase; MAP, migraine attack phase; NSAIDs, nonsteroidal anti-inflammatory drugs; oCNV, overall contingent negative variation; SE, standard error; tCNV, terminal contingent negative variation.
